# StaRProtein, A Web Server for Prediction of the Stability of Repeat Proteins

**DOI:** 10.1371/journal.pone.0119417

**Published:** 2015-03-25

**Authors:** Yongtao Xu, Xu Zhou, Meilan Huang

**Affiliations:** School of Chemistry and Chemical Engineering, Queen's University Belfast, David Keir Building, Stranmillis Road, Belfast, Northern Ireland, United Kingdom; Russian Academy of Sciences, Institute for Biological Instrumentation, RUSSIAN FEDERATION

## Abstract

Repeat proteins have become increasingly important due to their capability to bind to almost any proteins and the potential as alternative therapy to monoclonal antibodies. In the past decade repeat proteins have been designed to mediate specific protein-protein interactions. The tetratricopeptide and ankyrin repeat proteins are two classes of helical repeat proteins that form different binding pockets to accommodate various partners. It is important to understand the factors that define folding and stability of repeat proteins in order to prioritize the most stable designed repeat proteins to further explore their potential binding affinities. Here we developed distance-dependant statistical potentials using two classes of alpha-helical repeat proteins, tetratricopeptide and ankyrin repeat proteins respectively, and evaluated their efficiency in predicting the stability of repeat proteins. We demonstrated that the repeat-specific statistical potentials based on these two classes of repeat proteins showed paramount accuracy compared with non-specific statistical potentials in: 1) discriminate correct vs. incorrect models 2) rank the stability of designed repeat proteins. In particular, the statistical scores correlate closely with the equilibrium unfolding free energies of repeat proteins and therefore would serve as a novel tool in quickly prioritizing the designed repeat proteins with high stability. StaRProtein web server was developed for predicting the stability of repeat proteins.

## Introduction

Repeat protein scaffolds are commonly found in all kingdoms of life. They typically function in mediating specific protein-protein interactions which are essential for various biological functions [1]. Repeat proteins are comprised of tandem arrays of short repeat motifs that stack together to form extended super-helical structure. So far more than twenty classes of repeat proteins have been identified, among which the most abundant are ankyrin repeat (AR), leucine-rich repeat (LRR), armadillo repeat (ARM), helical-repeat (HEAT) and tetrotricopeptide repeat (TPR) proteins.

Repeat proteins are attractive alternative to antibodies due to their stability and ease of production as well as high binding affinities and specificity [2],[3]. In contrast to some repeat-containing proteins such as LRR and HEAT that bind a specific ligand with preferred secondary structure, TPR and AR proteins can bind with diverse proteins [4]. e.g. two discrete TPR domains in Hsp organizing protein (HOP) associate with molecular chaperone proteins Hsp70 and Hsp90, both being emerging cancer targets [5],[6],[7]. Envelope glyproteins, gp120 and gp41 medicate the entry of HIV-1 virus, and thus both are attractive anti-HIV targets [8]. Due to versatile binding profile of TPR and AR proteins, they can serve as useful scaffolds to mediate protein-protein interaction in biotechnology and therapeutics. Recently, a designed AR was developed to specifically recognize the surface glycoprotein gp120 as the inhibitor of HIV entry process and virus infection [9]. A stable consensus TPR protein was designed targeting HSP90 with mild affinity [10].

TPR and AR proteins are composed of repeating units of 34 and 33 amino acids, respectively. The basic repeat unit is helix-turn-helix turn in TPR and helix- β turn-helix-loop in AR protein.

Current protein engineering strategies mainly include structure-based rational design and sequence-based design such as directed evolution and consensus design. Consensus design of repeat proteins is focused on the consensus of individual repeats rather than the natural context in creating the templates. It would be useful to understand the structural nature of repeat proteins that define the folding and stability of designed proteins.

In the past two decades, knowledge-based statistical potentials was developed for protein folding and protein structure recognition [11], [12], [13] based on Anfinsen’s thermodynamics hypothesis [14]. Following the concept brought about by Sippl [12],[15], a variety of distance-dependent statistical potentials have been developed [16],[17],[18],[19],[20],[21],[22],[23]. The distance-dependant potential based on Boltzmann equation is given by:

$${ū}_{}\left(i,j,r\right)=-RTln\frac{N_{obs}^{}(i,j,r)}{N_{ref}^{}(i,j,r)}$$ (1)

Where R is the Boltzmann constant, T is the Kelvin temperature. N_obs_(i,j,r) is the observed number of atomic pairs (i, j) within a distance bin r in a database of experimental protein structures. N_ref_(i,j,r) is the reference state, which is the expected number of atomic atoms (i, j) in the same distance bin if there is no interaction between atoms.

The main difference of the statistical potentials lies in the selection of reference states. It was suggested that statistical potentials have a contradiction between the universality and pertinence and optimal reference states should be extracted based on specific application environment [24]. Statistical potential represents the pseudoenergy of proteins, therefore can be used to evaluate protein stability.

Unlike globular proteins, the stability of repeat proteins is dominant by the short-range interactions [25],[26]. Multistate kinetic folding pathway studies for some repeat proteins such as TPR and AR proteins disclosed that folding of these proteins is dominated by the competition between the stability of individual repeats and the interactions between repeats [25]. Pluckthun et. al. proposed that folding is a nucleation process, i.e. assembly of a minimal number of repeats triggers the entire folding process [27]. They suggested that the unfolding requires the progressive disruption of the folded repeat and therefore the stability is dependent on the number of repeats. Furthermore, it was suggested that all repeats in repeat proteins are not equal and different repeats have different contribution to stability [25],[28]. Therefore it is necessary to include sufficient features of repeat protein, e.g. distinct repeat proteins with low sequence identity and with different protein length, in the statistical potential libraries while calculating the distance-dependant statistical potentials. In order to evaluate the overall stability of repeat proteins and explore their application as novel binding molecules, we developed repeat-specific distance-dependant statistical potential libraries utilizing the structural features of two classes of helical repeat proteins TPR and AR. The structure-based statistical potential opens a way to evaluate the stability of the proteins that are designed by sequence-based approach, and can be used to quickly prioritize the proteins with predicted high stability for subsequent biological function exploitation.

## Materials and Methods

### All-atom distance-dependant statistical potentials

Distance-dependant statistical potentials are based on the assumption that the three-dimensional structure of a natural protein in its normal physiological environment has the lowest Gibbs free energy [14]. The stability of the proteins was evaluated by the all-atom probability discriminatory function (RAPDF) scoring function [17], which is based on conditional probability function representing preference of atomic distance.

$$P\left(C\right)*P{(d}_{ab}^{ij}|C)=P(d_{ab}^{ij})*P(C|d_{ab}^{ij})$$ (2)

where *P*(*C*): the probability that any structure picked at random is a member of the “correct” set.

$P{(d}_{ab}^{ij}|C)$: the probability of observing a distance d between two atoms *i* and *j* of types *a* and *b* in a correct structure.

$P(d_{ab}^{ij})$: the probability of observing such a distance in any structure, correct or incorrect.

$P(C|{\{d}_{ab}^{ij}\})$: the probability the structure is a member of the “correct” set, given it contains the distances $\{d_{ab}^{ij}\}.$

$\left\{d_{ab}^{ij}\right\}$ is the distance between atoms *i* and *j*, of type *a* and *b*, respectively.

The probabilities of observing the set of distances is expressed as products of the probabilities of observing each individual distance. An approximation is made that all distances are independent of one another, thus

$$P(\{d_{ab}^{ij}\}/C)=\prod_{ij}P(d_{ab}^{ij}/C);P(\{d_{ab}^{ij}\})=\prod_{ij}P\left(d_{ab}^{ij}\right)$$ (3)

From Equations (3) and (4), the following equation can be retrieved:

$$P{(C|\{d}_{ab}^{ij}\})=P\left(C\right)*\prod_{ij}\frac{P{(d}_{ab}^{ij}|C)}{P\left(d_{ab}^{ij}\right)}$$ (4)

Where P(C) is a constant independent of the conformation for a given amino acid sequence.

Statistical potential is obtained from statistics of experimental protein structures. All the atoms in the proteins are classified as 167 residue-specific heavy atom types [17] and the atomic distances between each atomic pair are calculated. These distances are then assigned to 18 different distance bins with distance cutoff value of 20 Å. Except for the first bin which is 0–3Å, the bin width of the rest of the bins is set as 1Å.

The score is given by the following logarithm of conditional probability:

$$S\left(\left\{d_{ab}^{ij}\right\}\right)=-\sum_{ij}ln\frac{P{(d}_{ab}^{ij}|C)}{P\left(d_{ab}^{ij}\right)}\propto -lnP{(C|\{d}_{ab}^{ij}\})$$ (5)

Here

$$P{(d}_{ab}^{ij}|C)=\frac{N_{obs}(i,j,r)}{N_{obs}(r)}$$ (6)

$$P\left(d_{ab}^{ij}\right)=\frac{N_{obs}(i,j)}{N_{total}}$$ (7)

Thus the scoring function becomes:

$$S\;(\left\{d_{ab}^{ij}\right\})=-\sum_{ij}\text{ln}\frac{\frac{N_{obs}\left(i,j,r\right)}{N_{obs}\left(r\right)}}{\frac{N_{obs}\left(i,j\right)}{N_{total}}}$$ (8)

N_obs_(i, j, *r*): The number of observed atomic pairs (i, j) of atomic type *a* and *b*, within bin *r*.

N_obs_(i, j): The number of observed atomic pairs (i, j) of atomic type *a* and *b*, within 18 bins.

N_obs_(*r*): The number of all observed atomic pairs within bin *r*.

N_total_: The number of all observed atomic pairs within 18 bins.

The statistical score of a particular protein is the sum of scores associated with all observed atomic pairs within 18 distance bins.

$$S_{score}=\sum_{ij}s_{ab}^{ij}$$ (9)

Where $s_{ab}^{i,j}$ is the statistical potential associated for atomic pairs (*i*, j) *with a value of*

$$-ln\frac{\frac{N_{obs}(i,j,r)}{N_{obs}(r)}}{\frac{N_{obs}(i,j)}{N_{total}}}.$$

### Database of reference protein structures

Six statistical libraries were constructed using α-, β-, α+β and general proteins, AR proteins and TPR proteins, respectively. The α-, β-, α+β and composite protein structure databases collected from Hobohm’s protein database [29]. The library of α+β protein structures was filtered by sequence identity cutoff of 25% and resolution cutoff of 1.5 Å resulting in 1271 proteins. The α- and β- protein collections were filtered by sequence identity cutoff of 25% and resolution cutoff of 3.5 Å, resulting in 1007 α- and 288 β- protein structures. The composite protein database is the sum of α-, β- and α+β databases. The original RAPDF potential based on a general protein database was also used to evaluate the stability of the proteins [17].

TPR and AR proteins were collected from SCOP [30] and PDB database. These proteins were filtered using sequence identity cutoff of 30% to construct the AR and TPR statistical library statistical libraries, which contain 33 AR proteins and 73 TPR proteins, respectively. PRIDE2 executable [31] was used to determine protein fold similarity and structural relationship was visualized using Drawtree and Drawgram functionalities in PHYLIP package (version 3.5c) [32]. The Arc of tree in Drawtree was set as 250°.

### Construction of decoy protein structures

Different decoy protein structures were collected or prepared to evaluate the efficiency of various statistical potential libraries on differentiation of correct structures from incorrect ones. Misfolded protein structures collected from the Decoy ‘R’ Us website were categorized into α-, β-, mixed α+β proteins and used as decoy structures [33]; for AR and TPR proteins, comparison was made between the natural proteins and their corresponding homology models. Additional comparison was made between designed consensus repeat proteins and their respective template scaffolds.

Homology models were built as decoy set for 8 proteins selected from the AR and TPR protein databases. The selection was made based on the criteria that there is sufficient sequence identity between the query and the template protein and they are evolutionary relevant species (sequence similarity is between 54% and 86%) (Table 1), thus the native and the decoy proteins have structural relevance. Homology models were built using Modeller (UCSF, USA) and the one with lowest DOPE score was kept for each protein.

**Table 1:** Template proteins used in homology modeling of repeat proteins.

Repeat protein	TPR pdb code	Natural resolution (Å)	No of repeats	Template pdb code	Template resolution (Å)	Identity (%)	RMSD
**TPR**	3EJN: A	1.50	Tpr like	4LER: A	1.42	54.1	1.133
	2C0M: A	2.50	8	4EQF: A	3.00	62.3	2.447
	3CEQ: B	2.75	5.5	3NF1: A	2.80	81.0	2.578
	3FP3: A	1.98	11	2GW1: A	3.00	58.5	2.414
	3SF4: A	2.60	8	4A1S: A	2.10	64.7	1.519
	3U84: A	2.50	3	3RE2: A	1.95	54.2	1.098
	4AM9: A	2.50	3	2XCB: A	1.85	61.8	1.027
	4GCO: A	1.60	3	2LNI: A	NMR	58.3	2.114
**Ankyrin**	1AWC: B	2.15	5	2P2C: P	3.24	69.9	1.193
	1BI7: B	3.40	4	1D9S: A	NMR	86.4	1.690
	1YCS: B	2.20	3	2VGE: A	2.10	59.2	1.071
	2ETB: A	1.65	6	2F37: A	1.70	86.2	1.020
	1YYH: A	1.90	6	1OT8: A	2.00	74.6	0.670
	3V30: A	1.57	5	3SO8: A	1.90	65.8	1.099
	1MJ0: A	2.03	5	2BKK: B	2.15	84.6	1.263
	1OT8: A	2.00	6	2F8Y: A	1.55	73.2	1.289

## Results and Discussion

### Statistical potentials based on general and α+β proteins

Recently, we evaluated the stability of self-derived peptides derived from three classes of envelope (E) proteins by two state-of-art statistical scoring functions, dDFIRE and RAPDF[17], [34]. It was found RAPDF based Monte Carlo selection method outperformed dDFIRE method for the beta-sheet Class II E proteins although both scoring functions display similar efficiency for the alpha-helical Class I HIV-1 gp41 and the mixed α+β Class III HSV-1 gB proteins [35]. Therefore in the current research, we developed statistical potential based on RAPDF.

**Fig 1:**
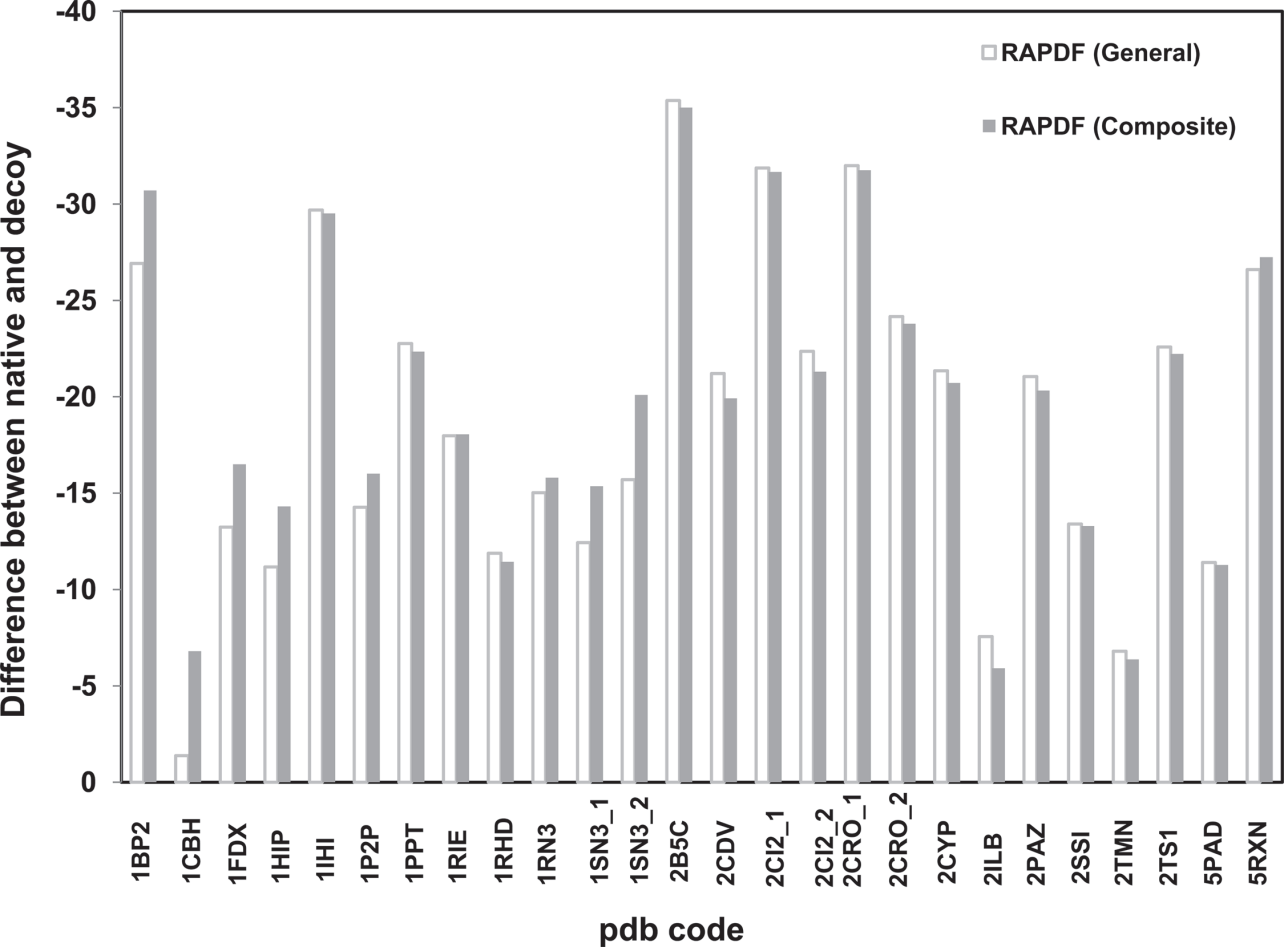
Evaluating the stability of general proteins using distance-dependant statistical potential based on *general protein library*. RAPDF (general) represents the statistical RAPDF scores calculated using the general protein database [17]. RAPDF (Composite) represents the statistical RAPDF scores calculated using the composite protein database composed of α-, β- and α+β proteins ***(2566 proteins)***.

**Fig 2:**
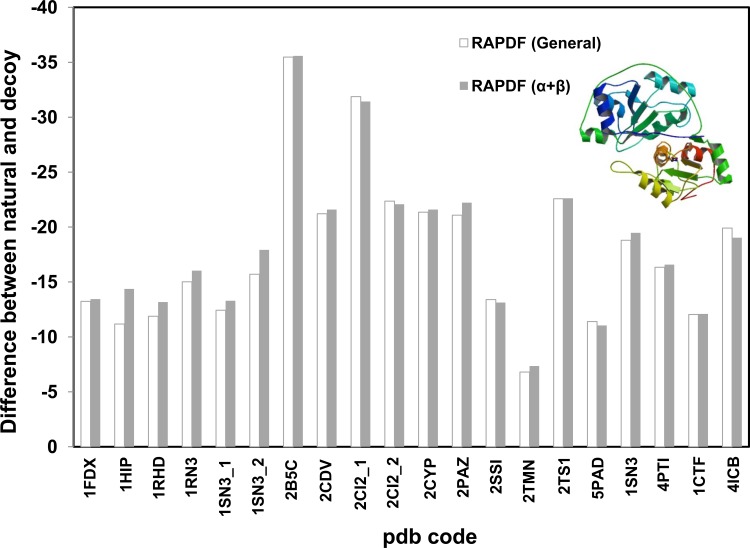
Evaluating the stability of α+β proteins using distance-dependant statistical potential based on *α+β protein library (1271 proteins)*. RAPDF (α+β) represents the statistical RAPDF scores calculated using the α- and β- databases. First 16 sets were single misfold decoy sets and the rest 4 sets were from multiple decoy sets with a representative decoy selected.

Statistical potential libraries based on α+β proteins as well as a composite database of 2566 proteins that comprises all α, β, and α+β proteins were constructed. 26 proteins and their misfolded decoy proteins were evaluated using the original RAPDF statistical potential and the potential based on the composite protein database. It was demonstrated that the score difference between the natural structures and their corresponding misfolded decoy structures is similar evaluated by these two general potentials (Fig. 1). Similarly, the stability score difference of 20 α+β proteins and their decoy partners is similar when it is evaluated using the statistical potential based on 1271 α+β protein and the original RAPDF potential (Fig. 2). It is not surprising as the statistical potentials of both the general protein and α+β databases were constructed based on a large dataset of protein structures such that the feature of common proteins was encompassed.

### Statistical potentials based on proteins with certain secondary structure

#### α and β statistical potentials

**Fig 3:**
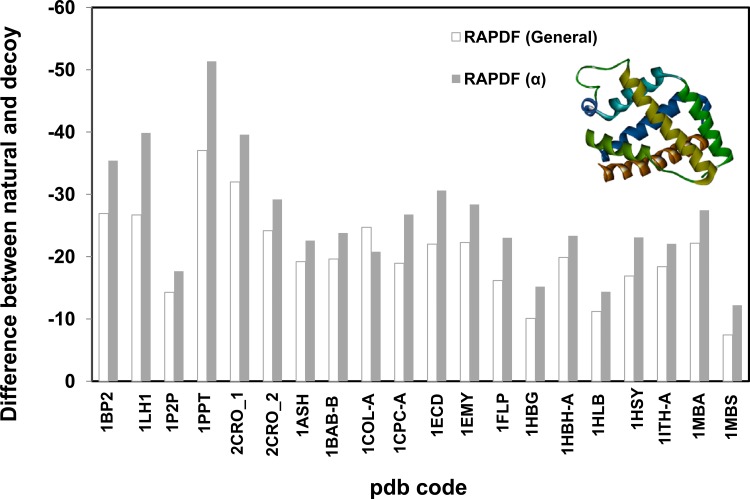
Evaluating the stability of α proteins using distance-dependant statistical potential based on α protein library (1007 proteins). RAPDF (α) represents the statistical RAPDF scores calculated using the α- database. First 6 sets were single misfold decoy sets and the rest 14 sets were from multiple decoy sets with a representative decoy selected.

**Fig 4:**
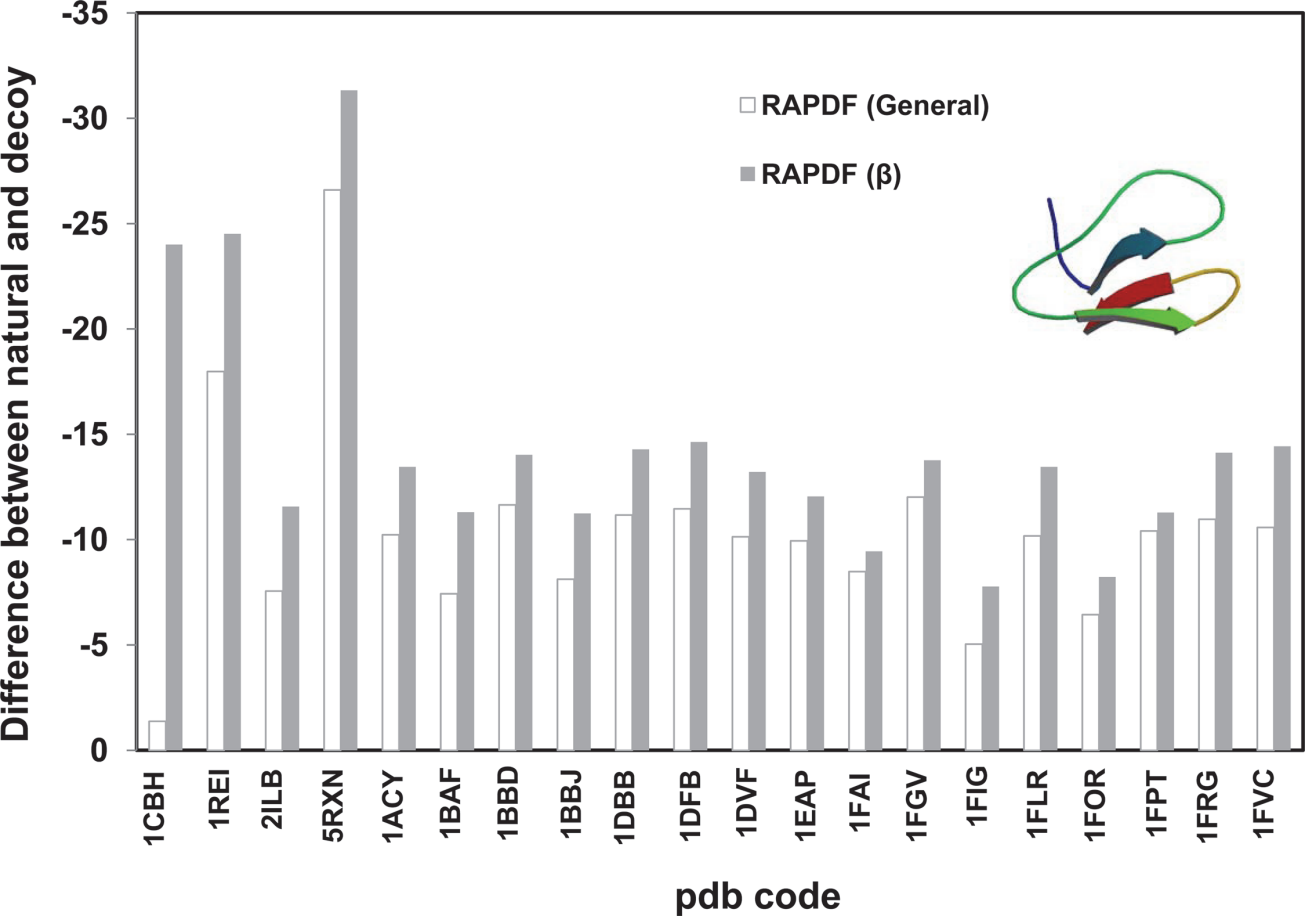
Evaluating the stability of β proteins using distance-dependant statistical potential based on β protein library (288 proteins). RAPDF (β) represents the statistical RAPDF scores calculated using the β- database. First 4 sets were single misfold decoy sets and the rest 16 sets were from multiple decoy sets with a representative decoy selected.

Since spatial arrangement of the atoms of proteins is crucial for distance-dependant statistical potential, we propose the feature of certain secondary structure should be reflected in the specific statistical libraries constructed based on representative protein secondary structures. Statistical potential libraries based on α-, β- proteins were constructed. The stability difference between the natural and misfolded decoy proteins is significantly larger when evaluated by the potentials constructed based on α or β proteins, compared with those evaluated by the general RAPDF potential (Figs. 3 and 4). The stability gap between the natural and incorrect structures is even greater for the dynamic solution structure of the C-terminal domain of cellobiohydrolase I (CT-CBH I), a β protein with two disulfide bonds (pdb code: 1CBH, Fig. 4) [36]. The general statistical potential is inferior to the β potential in identifying the correct conformation from the decoy one indicating the structural feature of the β-protein in particular the disulfide bridges is not sufficiently represented in the general potential library. We also evaluated multiple decoy sets collected from the Decoy ‘R’ Us website [33]. It can be seen that our method is also more effective in discriminating native or near-native from non-native ones (S1 Table).

Therefore it is necessary to use specific statistical potential to evaluate the stability of proteins with certain secondary structure.

#### Repeat-specific statistical potentials

100 TPR and 68 AR non-redundant proteins were collected from SCOP and PDB database. Using sequence identity cutoff of 30%, 33 AR proteins and 73 TPR and TPR-like proteins were retained to construct the AR- and TPR- specific statistical potentials. Although there are 8,000 AR sequences in the SMART database [37], only 33 AR proteins were identified with less than 30% sequence identity. This is because most of the resolved structures of AR were designed proteins which share high sequence similarity. The number of repeat or repeat-like motifs in the AR or TPR proteins is between 1 and 11.

**Fig 5:**
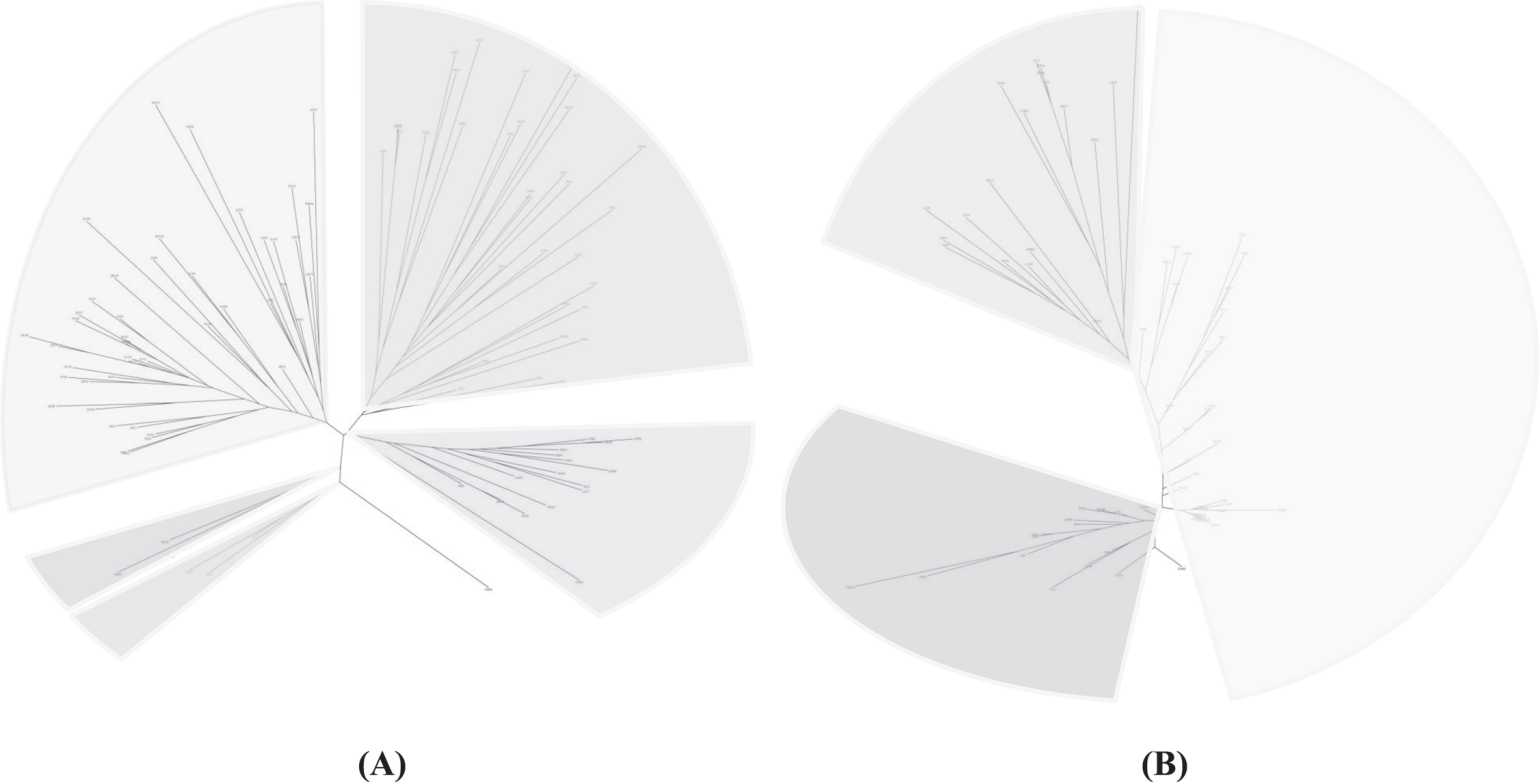
PRIDE2 structure comparison of non-redundant repeat proteins (Drawtree). The repeat proteins are divided into branches, which are shown as groups (A) AR (B) TPR.

Pair-wise protein fold similarity comparison was performed for the non-redundant TPR and AR protein database using PRIDE executables and the results were plotted using Drawtree (Fig. 5) and Drawgram (S1 Fig.). We found that the TPR protein library exhibits high diversity with the tree branches spreading around the origin. In contrast, the AR protein library is more populated, with a barren space, where no structure has been deposited. Structural comparison was also performed for the TPR and AR protein libraries filtered by 30% sequence similarity (S2 Fig. and S3 Fig.). Interestingly, compared with the TPR library, the proteins in the AR library are generally more similar in structure.

**Fig 6:**
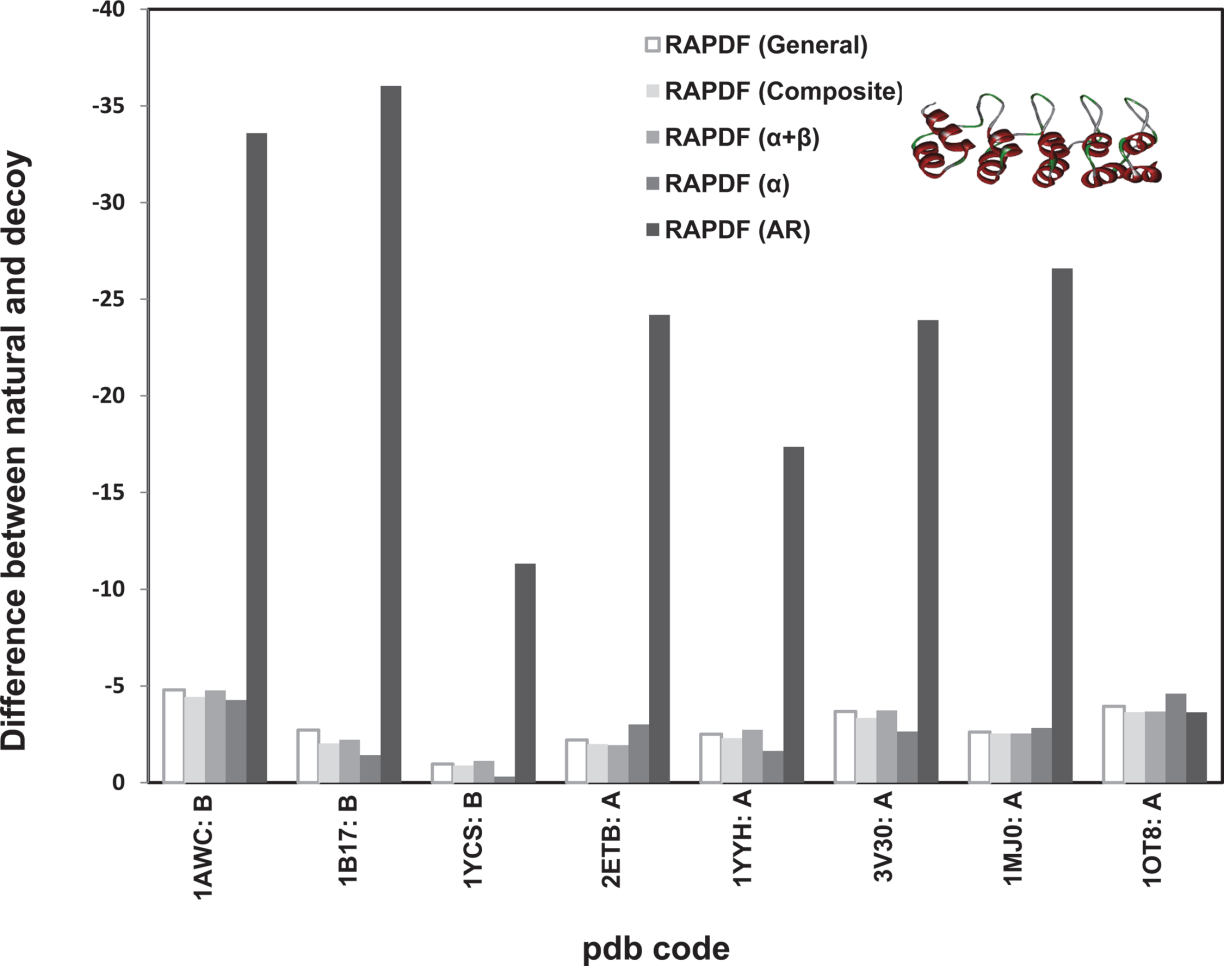
Distance-dependant statistical potential based on *ankyrin repeat protein library (33 proteins)*. Homology models were used as decoys. RAPDF (Ankyrin) represents the statistical RAPDF scores calculated using the Ankyrin database.

**Fig 7:**
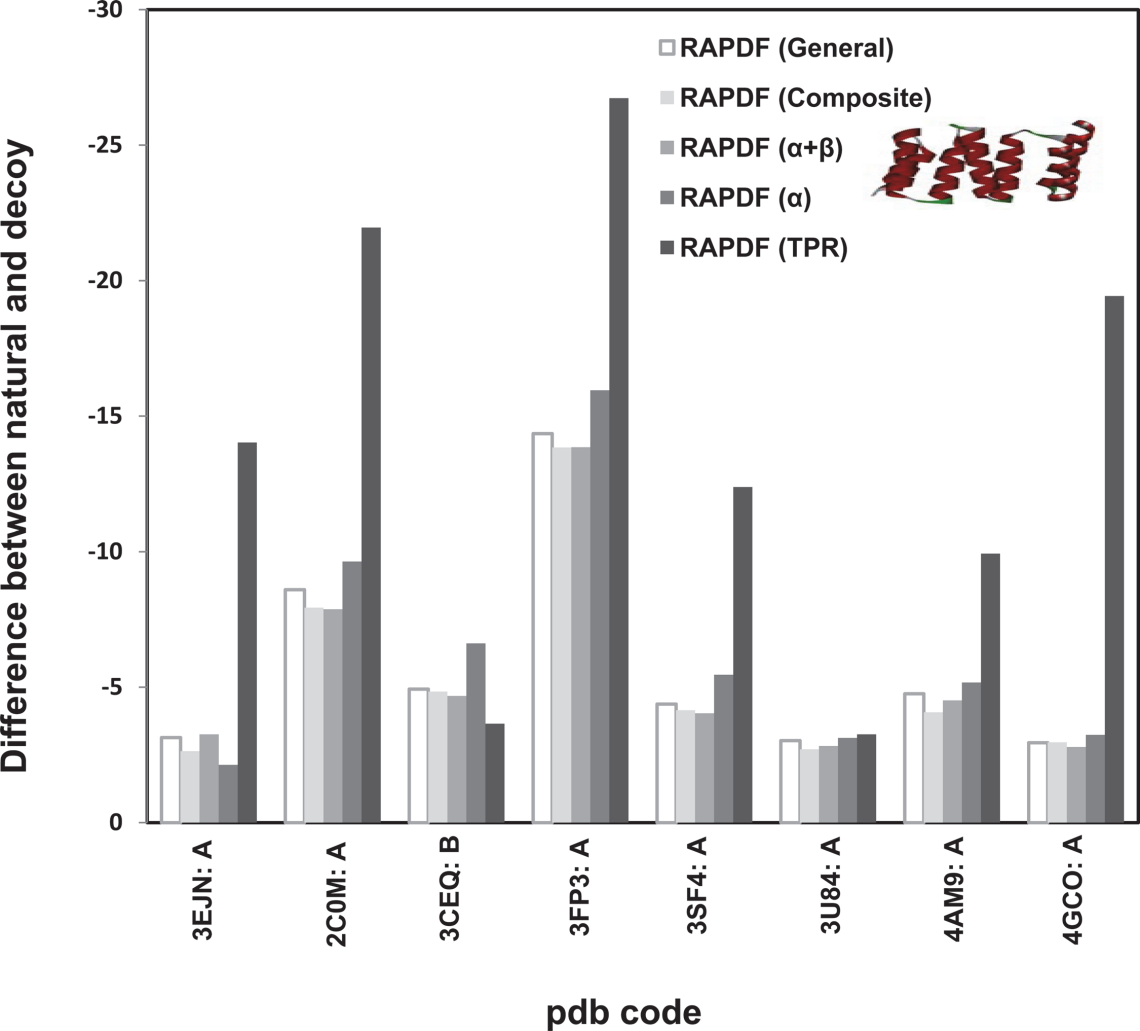
Distance-dependant statistical potential based on *TPR protein library (73 proteins)*. Homology models were used as decoys. RAPDF (TPR) represents the statistical RAPDF scores calculated using the TPR database.

Repeat-specific statistical libraries based on two classes of repeat proteins AR and TPR were constructed. Homology models for eight AR and eight TPR proteins were built as decoy structures and the stability difference between the natural proteins and the corresponding homology proteins were calculated using the repeat-specific statistical potentials (Figs. 6 and 7). We selected the templates which share similar sequence identify (54%-86%) to the natural ones to construct homology models as decoys such that they are structurally similar to the natural repeat (correct) proteins.

It was exhibited that the stability difference evaluated by AR or TPR specific statistical potential is remarkably higher than those evaluated by the general, α or β statistical potentials. This indicates the structural feature of the repeat proteins is sufficiently reflected in the statistical potential libraries and the repeat specific statistical potential is efficient in identifying natural repeat proteins from decoy structures even when the difference between the natural and decoy structures is trivial. It is worth noting that the stability difference is undetectable for the AR domain of Drosophila notch receptor (pdb code: 1OT8: A) [38] and two TPR proteins, the TPR domain of Human Kinesin Light Chain 2 (pdb code: 3CEQ: B) [39] and the TPR palm domain of Menin (pdb code: 3U84: A) [40]. This is because these repeat proteins have high structural similarity to their respective templates (S1 Fig.). In particular, the sequence identity between 3U84 and its template 3RE2 is only around 54% (Table 1), however, their statistical potential scores are indiscernible due to the exceptionally high structural similarity.

**Fig 8:**
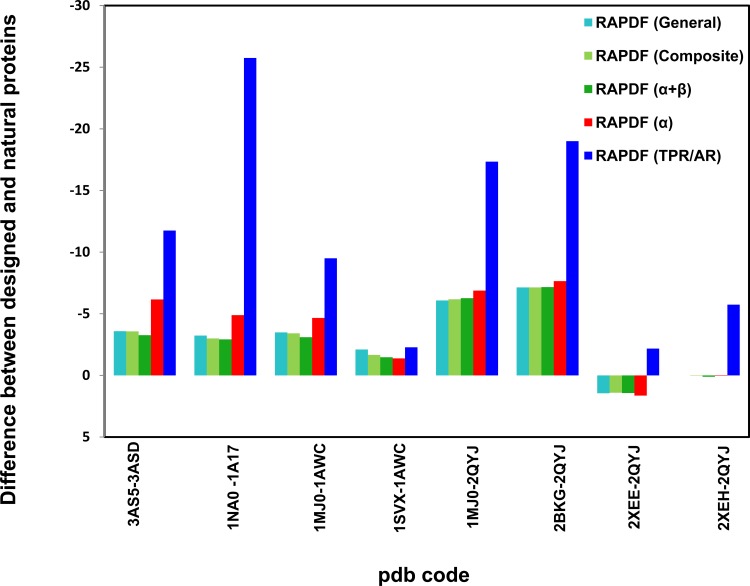
Predicted stability of designed repeat proteins using distance-dependant statistical potential based on TPR (light blue) or AR (blue) protein libraries.

Mutation of Arg50 of TPR-containing MamA protein (pdb code: 3AS5) into glutamate (pdb code: 3ASD) resulted in disruption of the salt bridge formed between Arg50 and Asp79 and destabilization of entire TPR1 of the protein [41]. We calculated the stability of the natural and mutant TPR proteins using the TPR-specific potential and found that the natural TPR is more stable than the mutant protein (Fig. 8). This is in agreement with the crystal structure of the R50E mutant, where the electron density for the TPR1was missing.

Due to the significance of repeat proteins in protein recognition, design of novel repeat proteins as alternative binding molecules to antibodies has become an attractive area in biotechnology. Consensus design is a useful biotechnology approach in constructing novel scaffolds to generate binding proteins with improved binding affinity and specificity.

In design of protein with desired binding activity, it is important to select a template onto which functional residues can be grafted. Consensus design is consensus construction of self-compatible repeat module template, a sequence of most frequent amino acid residues at each position decided by multiple sequence alignment. Two distinct consensus design strategies were used in design of AR and TPR proteins. Consensus AR proteins were constructed by fixing the conserved residues that maintain the repeat structures and randomizing the residues that are involved in target protein interaction [42],[43],[44]. In design of consensus TPR proteins, the repeat scaffold was modified by introducing functional residues involved in target protein recognition and specific binding.

The TPR-specific potential was used to evaluate the stability of consensus TPR proteins. CTPR3, a designed consensus TPR (pdb code: 1NA0) was reported to be more stable than the template protein phosphatase 5(PP5) (pdb code: 1P17) [45],[46]. Comparison of the statistical scores of the consensus TPR and the natural TPR manifested that the stability difference is more prominent than rest of the potentials (Fig. 7), in accordance with the experimental observation.

It was reported that the designed AR protein was more thermodynamic stable than the natural structure [42],[43]. The AR-specific potentials were used to evaluate the stability of designed consensus repeat proteins. Compared to the natural AR protein GABPβ1 (pdb code: 1AWC: B) [47], the designed consensus 5-repeat AR protein (E3_5) (pdb code: 1MJ0: A) [43] is associated with a lower statistical potential score, indicating it is more stable than the natural one (Fig. 6). The difference of the stability between the consensus and natural AR proteins is most prominent using the AR-specific potential among all the potentials, in accordance with the experimental observation.

Another consensus AR bound with maltose binding protein (MBP) (pdb code: 1SVX: A) is associated with comparable statistical score to that of the natural protein bound with GABPα [44]. Unlike TPR, LRR and WD40s proteins, AR and HEAT were reported to demonstrate great elasticity when binding with their targets [48],[49]. Thus the stability of AR in the bound complex is probably compromised by the conformational change when it binds to the target. Recently, it was reported the buried surface of protein is responsible for protein-protein binding affinity [50]. The buried surface area of consensus off7/ MBP is 611 Å^2^ [44], comparable to that of the natural AR protein in complex with GA binding protein (GABPα) (854 Å^2^) [47]. Thus the designed AR has similar binding affinity to the natural AR. In our previous study, we suggested that the structural stability of proteins is related to their *in situ* binding potential to the partner regions [35]. The off7 AR bound with MBP displayed comparable statistical score to that of the natural protein. This provides additional support to our assumption that the binding affinity of proteins is dependent on their stability.

E3_5 [43], E3_19 (pdb code, 2BKG) [51] and NI_3_C (pdb code: 2QYJ) [52] were designed AR proteins derived from same framework residues. E3_5 and E3_19 have difference sequences in that residues are different at randomized positions whileas NI_3_C has three full-consensus repeats. Our calculations demonstrated that NI_3_C has higher stability compared with E3_5 and E3_19. This is in line with observed high thermostability of NI_3_C, attributed to the increased salt-bridge interaction on its protein surface. NMR studies disclosed that unfolding of the C-terminal capping repeat limits the stability of designed ARs [53]. Two mutated forms of NI_3_C, NI_3_C_Mut5 (pdb code: 2XEE, where the C-terminus was extended by three residues) and NI_3_C_Mut6 (pdb code: 2XEH, where three additional charged residues were introduced to NI_3_C_Mut5) showed increased stability compared to the originally designed AR protein, attributed to increased buried surface area and additional salt-bridge or H-bond interactions [54]. The initially designed NI_3_C is already very stable and the two mutants are slightly more stable than NI_3_C. Using the statistical potential developed based on the AR proteins, we found both mutants are associated with higher RAPDF scores. In contrast, none of rest four statistical potentials could differentiate them.

### Comparison of statistical scores and equilibrium unfolding free energies

Unlike globular proteins, the stability of repeat proteins is dominated by short-range interactions [25], [26]. Folding kinetics indicated that there is a competition between the intrinsic stability of individual repeats and the interactions between repeats. Designed consensus repeat proteins have identical repeat units and therefore provide an excellent system for investigation of the thermodynamic properties of repeat proteins. Two series of TPR proteins, namely CTPR and CTPRa*n* proteins, which only differ by a double mutation per repeat, were engineered by the Regan and Main groups. The equilibrium unfolding and chemical unfolding of two series of CTPR proteins including seven proteins from the CTPRa*n* series (CTPRa*2* to CTPRa*10*) and two from the CTPR series (Table 2) were investigated. Among them, CTPR2 (pdb code: 1NA3) and CTPR3 have two and three 34-aa identical consensus repeats followed by a solvating helix [47]; CTPRa*8* (pdb code: 2AVP) contains eight TPR repeats [26].

**Table 2:** Comparison of kinetic energies and RAPDF scores of TPR proteins.

Protein	ΔG_D-N_ [54] (kcal/mol)	ΔG_0-j_ [55] (kcal/mol)	RAPDF (TPR)
**CTPRa*2***	3.2±0.6	3.3±0.9	−82.66
**CTPRa*3***	4.8±0.4	6.1±1.2	−127.68
**CTPRa*4***	4.2±1.1	9±1.5	−171.94
**CTPRa*5***	6±0.8	11.8±1.9	−217.1
**CTPRa*6***	7.7±0.2	14.7±2.2	−261.36
**CTPRa*8***	14.3±1.5	20.4±2.8	−350.81
**CTPRa*10***	23.4±2.1	26.1±3.5	−605.63
**CTPR*2***	7.6±1.1	-	−154.01
**CTPR*3***	12±0.7	-	−163.81

**Fig 9:**
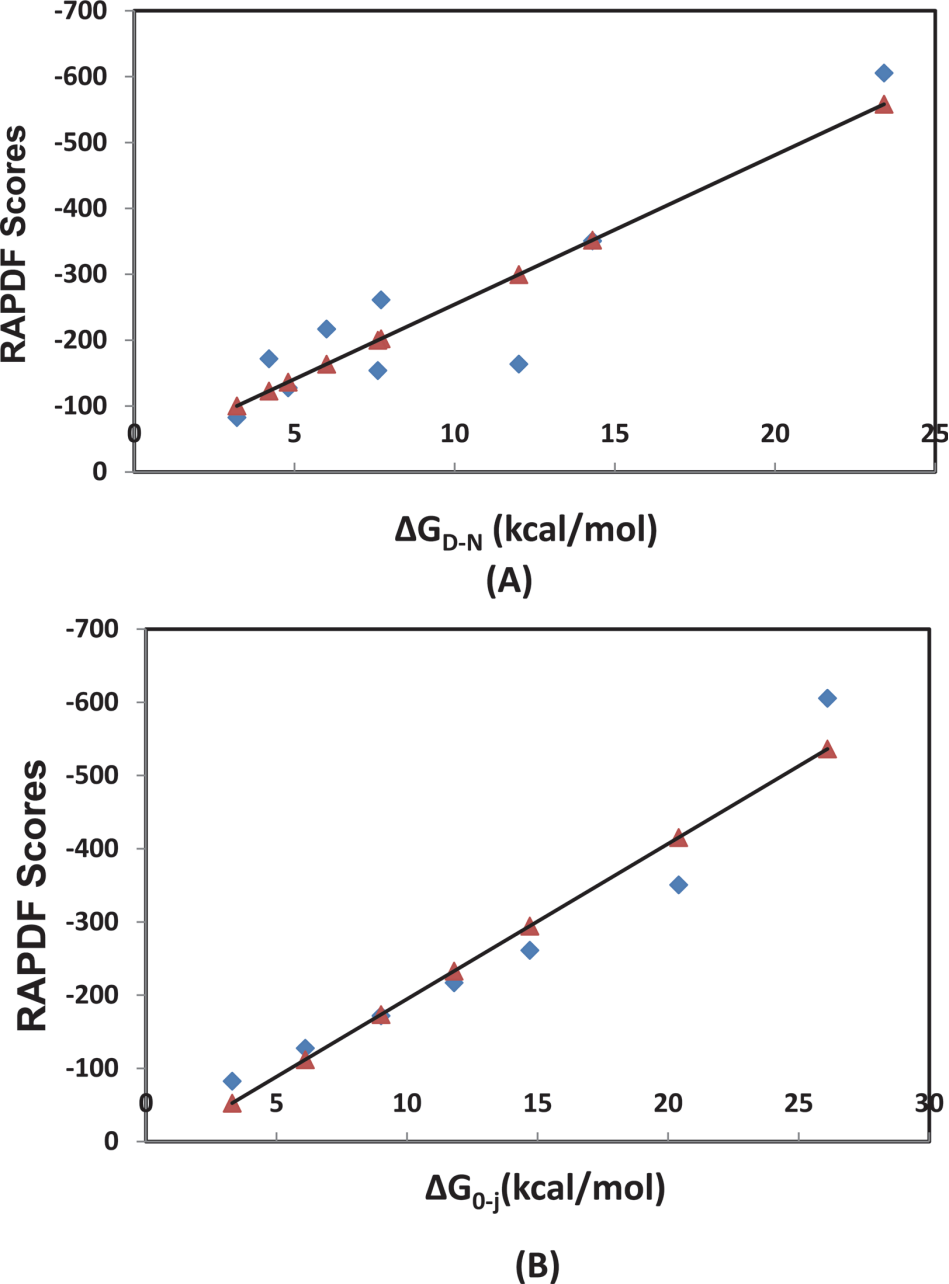
Correlation between the RAPDF scores of CTPRa*n* and the equilibrium unfolding free energies. (A) RAPDF scores versus ΔG_D-N_(kcal/mol), the thermal unfolding free energies (B) RAPDF scores versus ΔG_0-j_(kcal/mol), the folding free energies calculated from fitting the Ising model.

We calculated the stability of designed TPR proteins using the statistical potential and correlated the statistical scores with the thermal unfolding. The unfolding was monitored using differential scanning calorimetry (DSC) experiment and the model-independent free energies of unfolding (Δ*G*_D-N_) were calculated using the Gibbs-Helmholtz equation [55]. An obvious correlation was observed with a *R* ^2^ value of 0.84 (Fig. 9). Thermodynamic unfolding transition can be described by a 1D homozipper Ising model that treats each arrayed element of a repeat protein as an equivalent independently folding unit with nearest-neighbor pair-wise interactions between those units [26]. The free energies for folding were represented by Δ*G*0→j (j is the number of α-helices) [56]. We further correlated the statistical scores of CTPRa*n* with Δ*G*0→j that was calculated from fitting into the Ising model. A very strong correlation efficient *R* ^2^ of 0.93 was also observed. This is reasonable since the free energy is strongly correlated with the number of repeat units [25]. Whereas no correlation was found between the statistical scores and the unfolding energies for general globular proteins (S2 Table). The high correlation between the statistical scores and the equilibrium thermal/chemical unfolding free energies of repeat proteins suggests the statistical potential developed here can be accurately used to predict the stability of designed repeat proteins along the multistate kinetic folding pathways.

In consensus design or directed evolution, proteins are engineered so as to have admirable functions such as binding specificity or thermal stability. The designed libraries are usually large with the designed proteins being similar to the original scaffold. The statistical potential developed here can be used to quickly prioritize proteins in the libraries for subsequent functional assessment.

## Conclusions

Our research demonstrated that distance-dependant statistical potential is sensitive to the secondary structures. It is necessary to use the specific statistical potential based on specific protein secondary structure database to discriminate between correct and incorrect three-dimensional structures for a given sequence. We demonstrated that the repeat-specific statistical potentials we developed are efficient in differentiating the correct repeat protein structures from incorrect models. The statistical score correlate perfectly with equilibrium thermal/chemical unfolding free energy, and therefore would serve as a novel tool in quickly prioritizing designed repeat proteins with high stability.

The feature of repeat proteins allows for the evolution in biotechnology not only by mutation, but also by inserting, deleting, or shuffling the repeat motif, resulting in large combinatorial libraries. The repeat-specific distance-dependant statistical potentials can be used to rank stability of designed repeat proteins thus would provide guidance to prioritize repeat proteins from the designed combinatorial libraries based on their stability, in order to further explore their potential function in mediating protein-protein interactions.

A web server ‘Stability of Repeat Proteins’ (StaRProtein) is freely accessible via the URL http://StaRProtein.ch.qub.ac.uk. StaRProtein server is an on-line platform for evaluating protein stability, which is based on all-atom distance-dependant statistical potentials. Proteins with different secondary structures including alpha-, beta-, alpha+beta- and repeat proteins such as ankyrin repeat (AR) proteins and tetratricopeptide repeat (TPR) proteins are assessed using specific statistical potentials. Users can upload a protein structure in pdb format and designate the type of statistical potential library file. A statistical score which indicates the stability of the protein, the statistical potential library used and the length of the protein will be returned in output.

## Supporting Information

S1 Fig PRIDE2 structure comparison of non-redundant repeat proteins (Drawgram). The repeat proteins are divided into branches, which are shown as groups (A) AR (B) TPR.
(PDF)

S2 Fig PRIDE2 structure comparison of repeat proteins with less than 30% sequence identity (Drawtree). The repeat proteins are divided into branches, which are shown as groups (A) AR (B) TPR.
(PDF)

S3 Fig PRIDE2 structure comparison of repeat proteins with less than 30% sequence identity (Drawgram). The repeat proteins are divided into branches, which are shown as groups (A) AR (B) TPR.
(PDF)

S1 Table Statistical scores of multiple decoy proteins with different second structures. (PDF)

S2 Table Comparison of kinetic energies and RAPDF scores of globular proteins. (PDF)

## References

[1] Andrade MA, Perez-Iratxeta C, Ponting CP. Protein repeats: structures, functions, and evolution. J Struct Biol. 2001; 134:117–131. 11551174 10.1006/jsbi.2001.4392

[2] Suzuki F, Goto M, Sawa C, Ito S, Watanabe H, Sawada J, et al Functional interactions of transcription factor human GA-binding protein subunits. J Biol Chem. 1998; 273: 29302–29308. 9792629 10.1074/jbc.273.45.29302

[3] Malek S, Huxford T & Ghosh G. IκBα functions through direct contacts with the nuclear localization signals and the DNA binding sequences of NF-κB. J Biol Chem. 1998; 273: 25427–25435. 9738011 10.1074/jbc.273.39.25427

[4] Bork P. Hundreds of ankyrin-like repeats in functionally diverse proteins: mobile modules that cross phyla horizontally? Proteins: Struct Funct Genet. 1993; 17: 363–374. 8108379 10.1002/prot.340170405

[5] Evans CG, Chang L, Gestwicki JE. Heat shock protein 70 (hsp70) as an emerging drug target. J Med Chem. 2010; 53: 4585–4602. 10.1021/jm100054f 20334364 PMC2895966

[6] Dittmar KD, Demady DR, Stancato LF, Krishna P, Pratt WB. Folding of the glucocorticoid receptor by the heat shock protein (hsp) 90-based chaperone machinery. The role of p23 is to stabilize receptor.hsp90 heterocomplexes formed by hsp90.p60.hsp70. J Biol Chem. 1997; 272: 21213–21220. 9261129 10.1074/jbc.272.34.21213

[7] Morishima Y, Murphy PJ, Li DP, Sanchez ER, Pratt WB. Stepwise assembly of a glucocorticoid receptor.hsp90 heterocomplex resolves two sequential ATP-dependent events involving first hsp70 and then hsp90 in opening of the steroid binding pocket. J Biol Chem. 2000; 275:18054–18060. 10764743 10.1074/jbc.M000434200

[8] Teixeira C, Gomes JR, Gomes P, Maurel F, Barbault F. Viral surface glycoproteins, gp120 and gp41, as potential drug targets against HIV-1: brief overview one quarter of a century past the approval of zidovudine, the first anti-retroviral drug. Eur J Med Chem. 2011; 46:979–992. 10.1016/j.ejmech.2011.01.046 21345545

[9] Mann A, Friedrich N, Krarup A, Weber J, Stiegeler E, Dreier B, et al Conformation-dependent recognition of HIV gp120 by designed ankyrin repeat proteins provides access to novel HIV entry inhibitors. J Virol. 2013; 87: 5868–5881. 10.1128/JVI.00152-13 23487463 PMC3648163

[10] Cortajarena AL, Kajander T, Pan W, Cocco MJ, Regan L. Protein design to understand peptide ligand recognition by tetratricopeptide repeat proteins. Protein Eng Des & Sel. 2004; 17: 399–409. 10.1093/protein/gzh047 15166314

[11] Skolnick J, Jaroszewski L, Kolinski A, Godzik A. Derivation and testing of pair potentials for protein folding. When is the quasichemical approximation correct? Protein Sci. 1997; 6:676–688. 9070450 10.1002/pro.5560060317 PMC2143667

[12] Sippl MJ. Boltzmann’s principle, knowledge-based mean fields and protein folding. An approach to the computational determination of protein structures. J Computer-aided Mol Des. 1993; 7: 473–501. 8229096 10.1007/BF02337562

[13] Miyazawa S, Jernigan RL. Estimation of effective interresidue contact energies from protein crystal structures: quasi-chemical approximation. Macromolecules. 1985; 18: 534–552.

[14] Anfinsen CB. Principles that govern the folding of protein chains. Science. 1973; 181: 223–230. 4124164 10.1126/science.181.4096.223

[15] Sippl MJ. Calculation of conformational ensembles from potentials of mena force: an approach to the knowledge-based prediction of local structures in globular proteins. J Mol Biol. 1990; 213: 859–883. 2359125 10.1016/s0022-2836(05)80269-4

[16] Zhang C, Vasmatzis G, Cornette JL, DeLisi C. Determination of atomic desolvation energies from the structures of crystallized proteins. J Mol Biol. 1997; 267: 707–726. 9126848 10.1006/jmbi.1996.0859

[17] Samudrala R, Moult J. An all-atom distance-dependent conditional probability discriminatory function for protein structure prediction. J Mol Biol. 1998;275: 895–916. 9480776 10.1006/jmbi.1997.1479

[18] Lu H, Skolnick J. A distance-dependent atomic knowledge-based potential for improved protein structure selection. Proteins: Struct Funct Bioinfor. 2001; 44: 223–232. 11455595 10.1002/prot.1087

[19] Shen MY, Sali A. Statistical potential for assessment and prediction of protein structures. Protein Sci. 2006; 15: 2507–2524. 17075131 10.1110/ps.062416606 PMC2242414

[20] Zhou H, Zhou Y. Distance-scaled, finite ideal-gas reference state improves structure-derived potentials of mean force for structure selection and stability prediction. Protein Sci. 2002; 11: 2714–2726. 12381853 10.1110/ps.0217002 PMC2373736

[21] Rykunov D, Fiser A. Effects of amino acid composition, finite size of proteins, and sparse statistics on distance-dependent statistical pair potentials. Proteins: Struct Funct Bioinf. 2007; 67: 559–568. 17335003 10.1002/prot.21279

[22] Rykunov D, Fiser A. New statistical potential for quality assessment of protein models and a survey of energy functions. BMC Bioinformatics. 2010; 11:128 10.1186/1471-2105-11-128 20226048 PMC2853469

[23] Zhang J, Zhang Y. A novel side-chain orientation dependent potential derived from random-walk reference state for protein fold selection and structure prediction. PLoS ONE. 2010; 5:e15386 10.1371/journal.pone.0015386 21060880 PMC2965178

[24] Deng H, Jia Y, Wei Y, Zhang Y. What is the best reference state for designing statistical atomic potentials in protein structure prediction? Proteins: Struct Funct Bioinf. 2012; 80:2311–2322. 10.1002/prot.24121 22623012 PMC3409322

[25] Mello CC and Barrick D, An experimentally determined protein folding energy landscape. Proc Natl Acad Sci U S A. 2004; 101: 14102–14107. 15377792 10.1073/pnas.0403386101 PMC521126

[26] Kajander T, Cortajarena AL, Main ER, Mochrie SG, Regan L. A new folding paradigm for repeat proteins. J Am Chem Soc. 2005; 127:10188–90. 16028928 10.1021/ja0524494

[27] Wetzel SK, Settanni G, Kenig M, Binz HK, Pluckthun A. Folding and unfolding mechanism of highly stable full-consensus ankyrin repeat proteins. J Mol Biol. 2008; 376: 241–257. 10.1016/j.jmb.2007.11.046 18164721

[28] Zhang B. & Peng Z. A minimum folding unit in the ankyrin repeat protein p16INK4. J Mol Biol. 2000; 299:1121–1132. 10843863 10.1006/jmbi.2000.3803

[29] Griep S, Hobohm U. PDBselect 1992–2009 and PDBfilter-select. Nucleic Acids Res. 2010; 38(Database issue): D318–319. 10.1093/nar/gkp786 19783827 PMC2808879

[30] Murzin AG, Brenner SE, Hubbard T, Chothia C. SCOP: a structural classification of proteins database for the investigation of sequences and structures. J Mol Biol. 1995; 247: 536–540. 7723011 10.1006/jmbi.1995.0159

[31] Gáspári Z, Vlahovicek K, Pongor S. Efficient recognition of folds in protein 3D structures by the improved PRIDE algorithm. Bioinformatics. 2005; 21:3322–3323. 15914542 10.1093/bioinformatics/bti513

[32] Felsenstein J. PHYLIP (Phylogeny Inference Package) version 3.5c Department of Genetics, University of Washington, Seattle 1993; Accessed 27 October 2014.

[33] Samudrala R, Levitt M. Decoys 'R' Us: A database of incorrect protein conformations to improve protein structure prediction. Protein Science. 2000; 9: 1399–1401. 10933507 10.1110/ps.9.7.1399 PMC2144680

[34] Yang Y, Zhou Y. Specific interactions for ab initio folding of protein terminal regions with secondary structures. Proteins Struct Funct Bioinf. 2008; 72:793–803. 10.1002/prot.21968 18260109

[35] Xu Y, Rahman NA, Othman R, Hu P, Huang M. Computational identification of self-inhibitory peptides from envelope proteins. Proteins: Struct Funct Bioinf. 2012; 80: 2154–2168. 10.1002/prot.24105 22544824

[36] Kraulis J, Clore GM, Nilges M, Jones TA, Pettersson G, Knowles J, et al Determination of the three-dimensional solution structure of the C-terminal domain of cellobiohydrolase I from Trichoderma reesei. A study using nuclear magnetic resonance and hybrid distance geometry-dynamical simulated annealing. Biochemistry. 1989; 28:7241–7257. 2554967 10.1021/bi00444a016

[37] Letunic I, Goodstadt L, Dickens NJ, Doerks T, Schultz J, Mott R, Ciccarelli F, et al Recent improvements to the SMART domain-based sequence annotation resource. Nucleic Acids Res. 2002; 30: 242–244. 11752305 10.1093/nar/30.1.242 PMC99073

[38] Zweifel ME, Leahy DJ, Hughson FM, Barrick D. Structure and stability of the ankyrin domain of the Drosophila Notch receptor. Protein Sci. 2003; 12: 2622–2632. 14573873 10.1110/ps.03279003 PMC2366946

[39] Zhu H, Lee HY, Tong Y, Hong BS, Kim KP, Shen Y, et al Crystal Structures of the Tetratricopeptide Repeat Domains of Kinesin Light Chains: Insight into Cargo Recognition Mechanisms. PLoS ONE. 2012; 7: e33943 10.1371/journal.pone.0033943 22470497 PMC3314626

[40] Huang J, Gurung B, Wan B, Matkar S, Veniaminova NA, Wan K, et al The same pocket in menin binds both MLL and JUND but has opposite effects on transcription. Nature. 2012; 482: 542–546. 10.1038/nature10806 22327296 PMC3983792

[41] Zeytuni N, Ozyamak E, Ben-Harush K, Davidov G, Levin M, Gat Y, et al Self-recognition mechanism of MamA, a magnetosome-associated TPR-containing protein, promotes complex assembly. Proc Natl Acad Sci U S A. 2011; 108: E480–487. 10.1073/pnas.1103367108 21784982 PMC3158213

[42] Binz HK, Stumpp MT, Forrer P, Amstutz P & Plückthun A. Designing repeat proteins: well-expressed, soluble and stable proteins from combinatorial libraries of consensus ankyrin repeat proteins. J Mol Biol. 2003; 332: 489–503. 12948497 10.1016/s0022-2836(03)00896-9

[43] Kohl A, Binz HK, Forrer P, Stumpp MT, Plückthun A, Grütter MG. Designed to be stable: crystal structure of a consensus ankyrin repeat protein. Proc Natl Acad Sci U S A. 2003; 100:1700–1705. 12566564 10.1073/pnas.0337680100 PMC149896

[44] Binz HK, Amstutz P, Kohl A, Stumpp MT, Briand C, Forrer P, et al High-affinity binders selected from designed ankyrin repeat protein libraries. Nat Biotechnol. 2004; 22: 575–582. 15097997 10.1038/nbt962

[45] Canyuk B, Medrano FJ, Wenck MA, Focia PJ, Eakin AE, Craig SP 3rd.Interactions at the dimer interface influence the relative efficiencies for purine nucleotide synthesis and pyrophosphorolysis in a phosphoribosyltransferase. J Mol Biol. 2004;3354:905–21. 10.1016/j.jmb.2003.11.012 14698288

[46] Main ER, Xiong Y, Cocco MJ, D'Andrea L, Regan L. Design of stable alpha-helical arrays from an idealized TPR motif. Structure. 2003; 11: 497–508. 12737816 10.1016/s0969-2126(03)00076-5

[47] Batchelor AH, Piper DE, de la Brousse FC, McKnight SL, Wolberger C. The structure of GABPalpha/beta: an ETS domain- ankyrin repeat heterodimer bound to DNA. Science. 1998; 279: 1037–1041. 9461436 10.1126/science.279.5353.1037

[48] Grove TZ, Cortajarena AL, Regan L. Ligand binding by repeat proteins: natural and designed. Curr Opin Struct Biol. 2008; 18: 507–515. 10.1016/j.sbi.2008.05.008 18602006 PMC3500881

[49] Lee G, Abdi K, Jiang Y, Michaely P, Bennett V, Marszalek PE. Nanospring behaviour of ankyrin repeats. Nature. 2006; 440: 246–249. 16415852 10.1038/nature04437

[50] Chen J, Sawyer N, Regan L. Protein-protein interactions: general trends in the relationship between binding affinity and interfacial buried surface area. Protein Sci. 2013; 22: 510–515. 10.1002/pro.2230 23389845 PMC3610057

[51] Binz HK, Kohl A, Pluckthun A, Grutter MG. Crystal structure of a consensus-designed ankyrin repeat protein: implications for stability. Proteins: Struct Funct Bioinf. 2006; 65: 280–284. 10.1002/prot.20930 16493627

[52] Merz T, Wetzel SK, Firbank S, Plückthun A, Grütter MG, Mittl PR. Stabilizing ionic interactions in a full-consensus ankyrin repeat protein. J Mol Biol. 2008; 376:232–40. 18155045 10.1016/j.jmb.2007.11.047

[53] Wetzel SK, Ewald C, Settanni G, Jurt S, Plückthun A, Zerbe O. Residue-resolved stability of full-consensus ankyrin repeat proteins probed by NMR. J. Mol Biol. 2010; 402: 241–258. 10.1016/j.jmb.2010.07.031 20654623

[54] Kramer M, Wetzel SK, Pluckthun A, Mittl P, Grutter M. Structural determinants for improved stability of designed ankyrin repeat proteins with a redesigned C-capping module. J Mol Biol. 2010; 404: 381–391. 10.1016/j.jmb.2010.09.023 20851127

[55] Phillips JJ, Javadi Y, Millership C, Main ER. Modulation of the multistate folding of designed TPR proteins through intrinsic and extrinsic factors. Protein Sci. 2012; 21:327–338. 10.1002/pro.2018 22170589 PMC3375434

[56] Javadi Y and Main ERG. Exploring the folding energy landscape of a series of designed consensus tetratricopeptide repeat proteins. Proc Natl Acad Sci U S A. 2009; 106:17383–17388. 10.1073/pnas.0907455106 19805120 PMC2765091

