# Supplementary material for: StaRProtein, A Web Server for Prediction of the Stability of Repeat Proteins
Source: PLoS One. 2015 Mar 25;10(3):e0119417. doi: 10.1371/journal.pone.0119417 (PMC4373711; doi:10.1371/journal.pone.0119417)
Supplement: S1 Fig — The repeat proteins are divided into branches, which are shown as groups (A) AR (B) TPR. (PDF) [file pone.0119417.s001.pdf]

S1 Figure. PRIDE2 structure comparison of non-redundant repeat proteins (Drawgram).

The repeat proteins are divided into branches, which are shown as groups (A) AR (B) TPR.

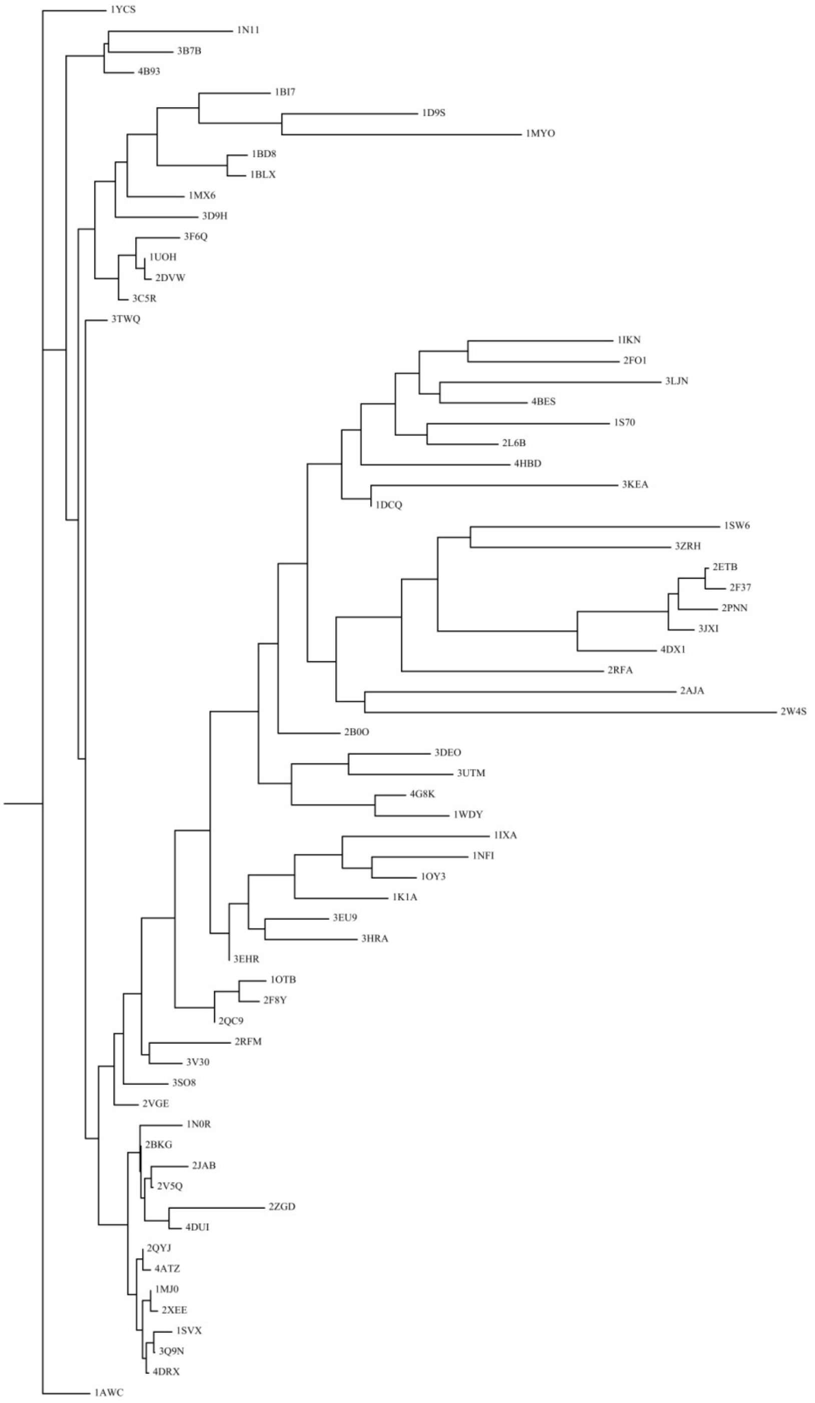
1YCS
1N11
3B7B
4B93
1BI7
1D9S
1MYO
1BD8
1BLX
1MX6
3D9H
3F6Q
1UOH
2DVW
3C5R
3TWQ
1IKN
2FO1
3LJN
4BES
1S70
2L6B
4HBD
3KEA
1DCQ
1SW6
3ZRH
2ETB
2F37
2PNN
3JXI
4DX1
2RFA
2AJA
2W4S
2B0O
3DEO
3UTM
4G8K
1WDY
1IXA
1NFI
1OY3
1K1A
3EU9
3HRA
3EHR
1OTB
2F8Y
2QC9
2RFM
3V30
3SO8
2VGE
1N0R
2BKG
2JAB
2V5Q
2ZGD
4DUI
2QYJ
4ATZ
1MJ0
2XEE
1SVX
3Q9N
4DRX
1AWC

(A)

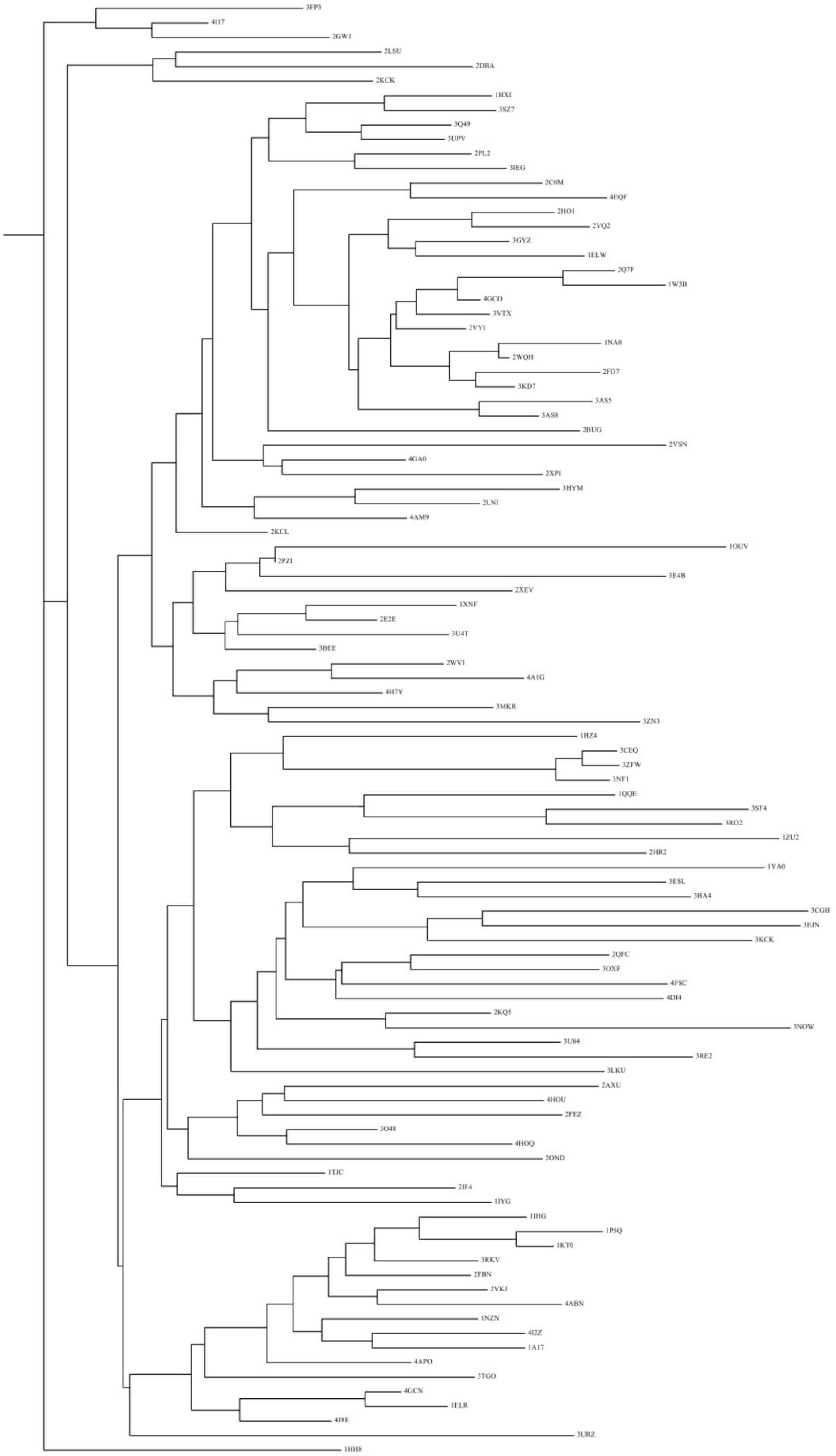
3FP3
4I17
2GW1
2LSU
2DBA
2KCK
1HXI
3SZ7
3Q49
3UPV
2PL2
3IEG
2C0M
4EQF
2HO1
2VQ2
3GYZ
1ELW
2Q7F
1W3B
4GCO
3VTX
2VYI
1NA0
2WQH
2FO7
3KD7
3AS5
3AS8
2BUG
2VSN
4GA0
2XPI
3HYM
2LNI
4AM9
2KCL
1OUV
2PZI
3E4B
2XEV
1XNF
2E2E
3U4T
3BEE
2WVI
4A1G
4H7Y
3MKR
3ZN3
1HZ4
3CEQ
3ZFW
3NF1
1QQE
3SF4
3RO2
1ZU2
2HR2
1YA0
3ESL
3HA4
3CGH
3EJN
3KCK
2QFC
3OXF
4FSC
4DI4
2KQ5
3NOW
3U84
3RE2
3LKU
2AXU
4HOU
2FEZ
3O48
4HOQ
2OND
1TJC
2IF4
1IYG
1IHG
1P5Q
1KT0
3RKV
2FBN
2VKJ
4ABN
1NZN
4I2Z
1A17
4APO
3TGO
4GCN
1ELR
4J8E
3URZ
1HH8

(B)
