# Supplementary material for: StaRProtein, A Web Server for Prediction of the Stability of Repeat Proteins
Source: PLoS One. 2015 Mar 25;10(3):e0119417. doi: 10.1371/journal.pone.0119417 (PMC4373711; doi:10.1371/journal.pone.0119417)
Supplement: S2 Fig — The repeat proteins are divided into branches, which are shown as groups (A) AR (B) TPR. (PDF) [file pone.0119417.s002.pdf]

S2 Figure. PRIDE2 structure comparison of repeat proteins with less than 30% sequence identity (Drawtree). The repeat proteins are divided into branches, which are shown as groups (A) AR (B) TPR.

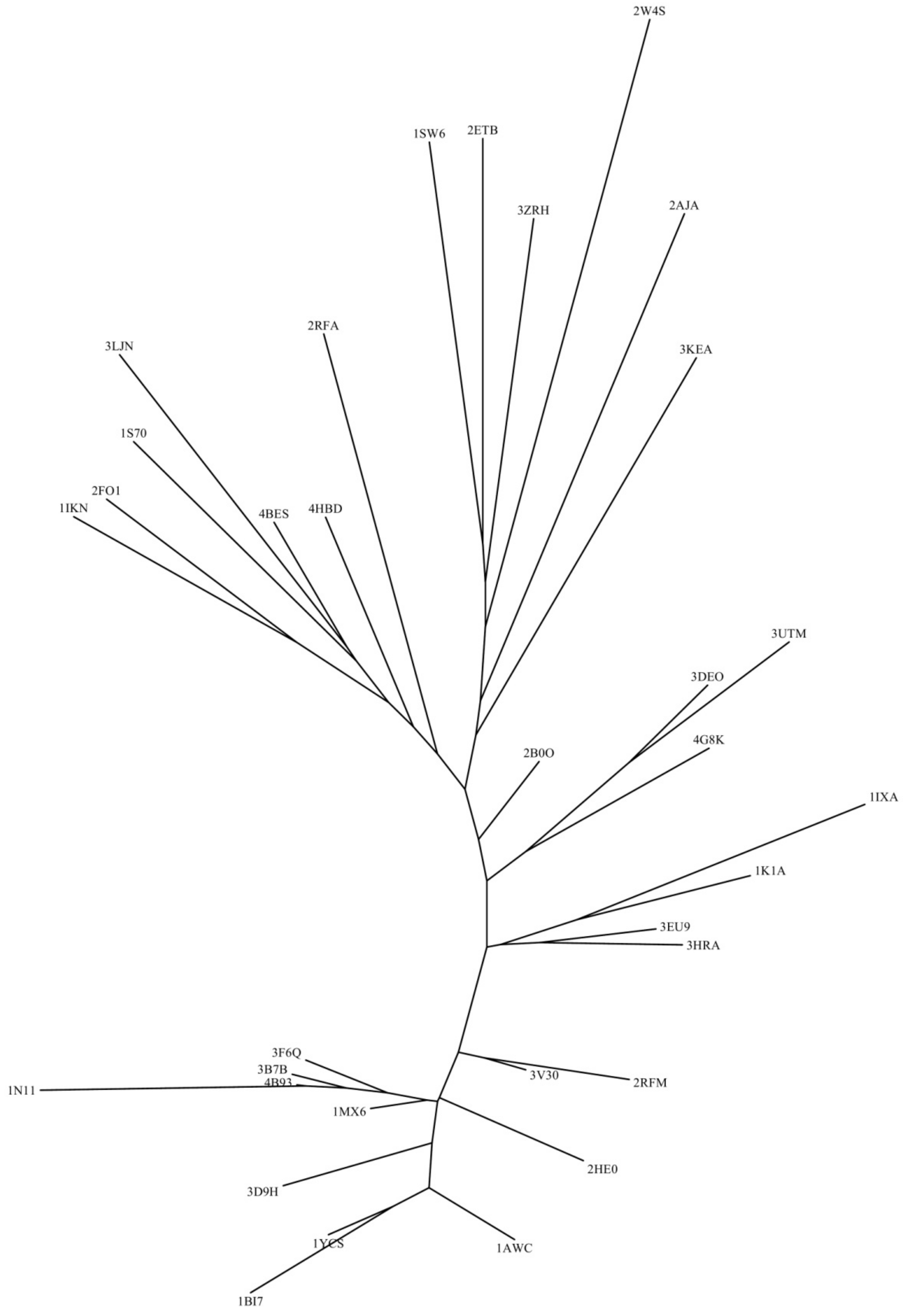
2W4S
1SW6
2ETB
3ZRH
2AJA
2RFA
3LJN
3KEA
1S70
2FO1
1IKN
4BES
4HBD
3UTM
3DEO
4G8K
2B0O
1IXA
1K1A
3EU9
3HRA
3F6Q
3B7B
4B93
1N11
1MX6
3V30
2RFM
2HE0
3D9H
1YCS
1AWC
1BI7

(A)

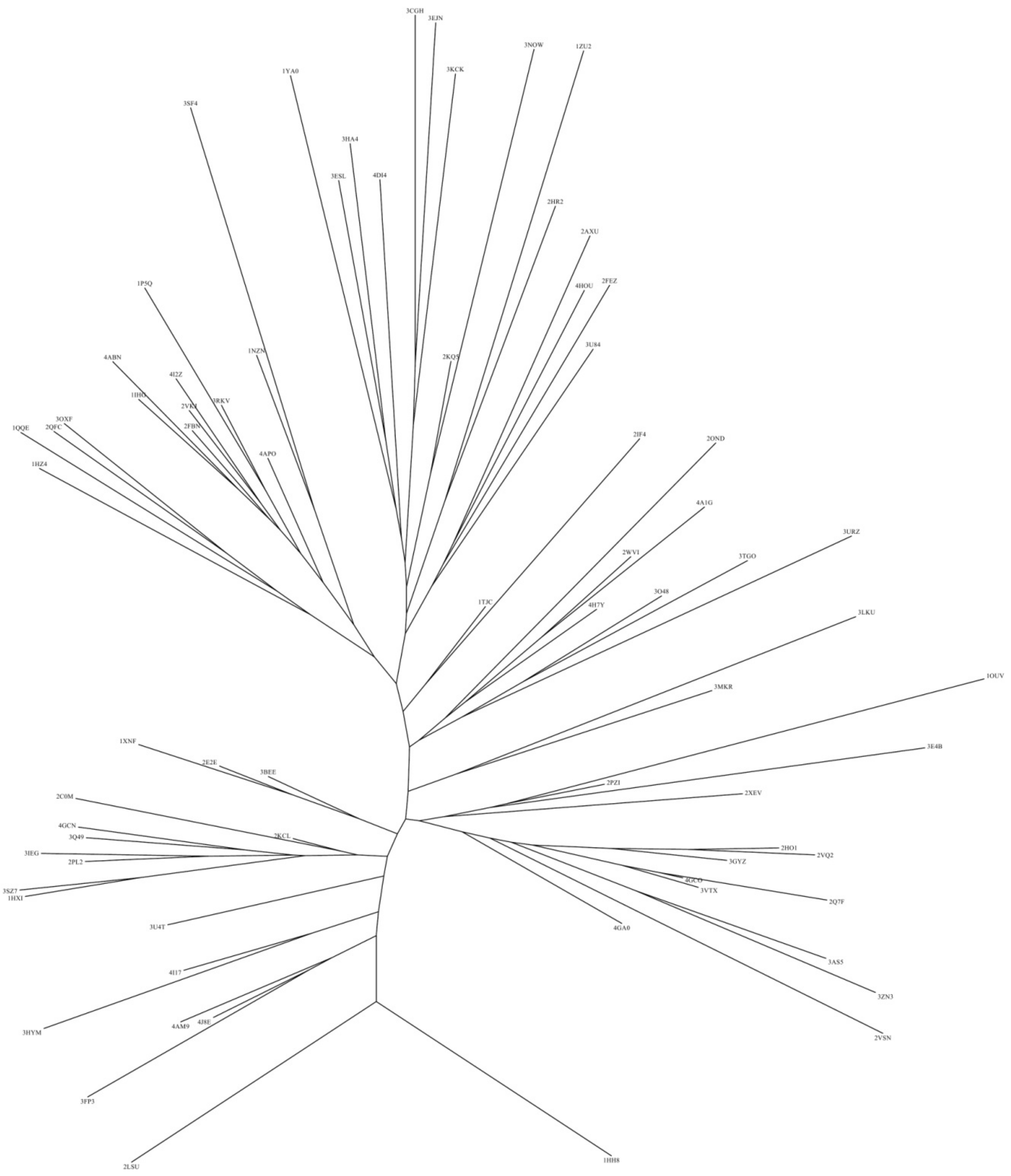
3CGH
3EJN
3NOW
1ZU2
1YA0
3KCK
3SF4
3HA4
3ESL
4DI4
2HR2
2AXU
4HOU
2FEZ
1P5Q
3U84
1NZN
2KQS
4ABN
4I2Z
1IHO
2VEI
3RKV
3OXF
2FBN
2QFC
1QQE
4APO
1HZ4
2IF4
2OND
4A1G
3URZ
2WVI
3TGO
1TJC
4H7Y
3O48
3LKU
1OUV
3MKR
1XNF
3E4B
2E2E
3BEE
2PZI
2XEV
2C0M
4GCN
2KCL
3Q49
2HO1
3IEG
2VQ2
2PL2
3GYZ
4GCO
3VTX
3SZ7
1HXI
2Q7F
3U4T
4GA0
3AS5
4I17
3ZN3
3HYM
4AM9
4J8E
2VSN
3FP3
2LSU
1HH8

(B)
