# Supplementary material for: StaRProtein, A Web Server for Prediction of the Stability of Repeat Proteins
Source: PLoS One. 2015 Mar 25;10(3):e0119417. doi: 10.1371/journal.pone.0119417 (PMC4373711; doi:10.1371/journal.pone.0119417)
Supplement: S3 Fig — The repeat proteins are divided into branches, which are shown as groups (A) AR (B) TPR. (PDF) [file pone.0119417.s003.pdf]

S3 Figure. PRIDE2 structure comparison of repeat proteins with less than 30% sequence identity (Drawgram). The repeat proteins are divided into branches, which are shown as groups (A) AR (B) TPR.

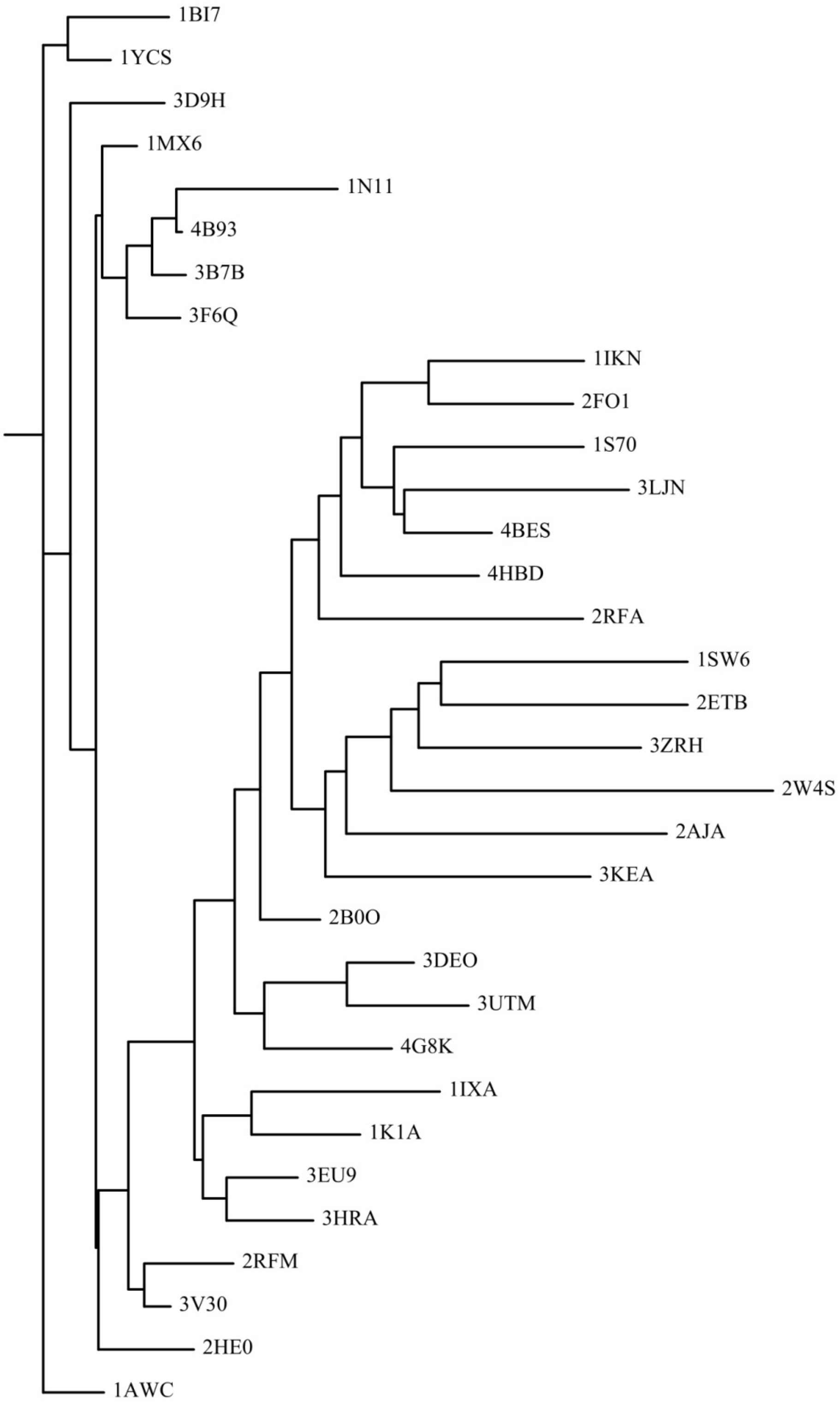
1BI7
1YCS
3D9H
1MX6
1N11
4B93
3B7B
3F6Q
1IKN
2FO1
1S70
3LJN
4BES
4HBD
2RFA
1SW6
2ETB
3ZRH
2W4S
2AJA
3KEA
2B0O
3DEO
3UTM
4G8K
1IXA
1K1A
3EU9
3HRA
2RFM
3V30
2HE0
1AWC

(A)

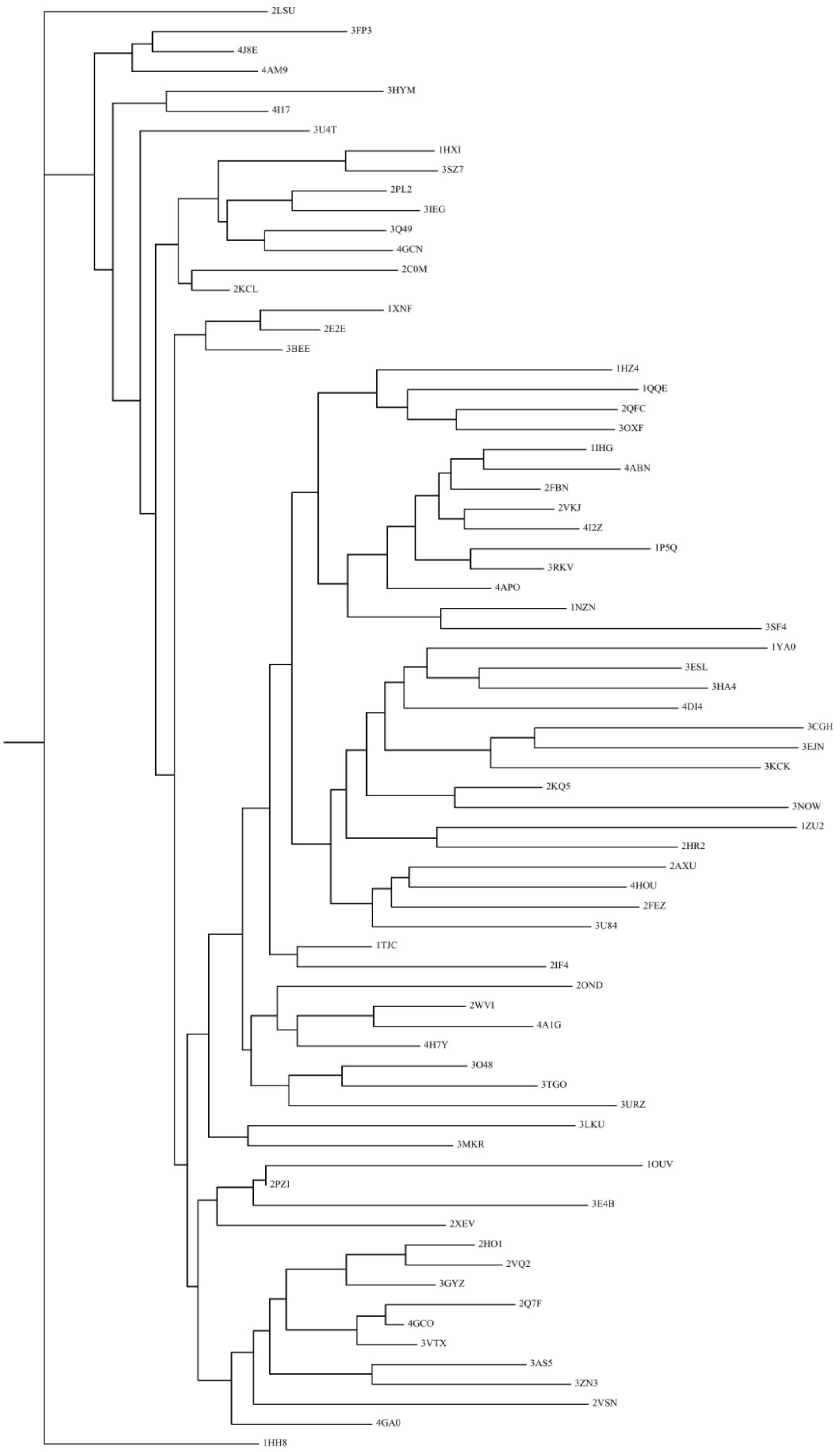
2LSU
3FP3
4J8E
4AM9
3HYM
4I17
3U4T
1HXI
3SZ7
2PL2
3IEG
3Q49
4GCN
2C0M
2KCL
1XNF
2E2E
3BEE
1HZ4
1QQE
2QFC
3OXF
1IHG
4ABN
2FBN
2VKJ
4I2Z
1P5Q
3RKV
4APO
1NZN
3SF4
1YA0
3ESL
3HA4
4DI4
3CGH
3EJN
3KCK
2KQ5
3NOW
1ZU2
2HR2
2AXU
4HOU
2FEZ
3U84
1TJC
2IF4
2OND
2WVI
4A1G
4H7Y
3O48
3TGO
3URZ
3LKU
3MKR
1OUV
2PZI
3E4B
2XEV
2HO1
2VQ2
3GYZ
2Q7F
4GCO
3VTX
3AS5
3ZN3
2VSN
4GA0
1HH8

(B)
