# Supplementary material for: StaRProtein, A Web Server for Prediction of the Stability of Repeat Proteins
Source: PLoS One. 2015 Mar 25;10(3):e0119417. doi: 10.1371/journal.pone.0119417 (PMC4373711; doi:10.1371/journal.pone.0119417)
Supplement: S1 Table — (PDF) [file pone.0119417.s004.pdf]

**S1 Table**

**Statistical scores of multiple decoy proteins with different second structures.**

| Natives | Decoys[1] | RMSD | $Gen_{Native}$ | $Gen_{Decoy}$ | ΔGen | $\alpha_{Native}$ | $\alpha_{Decoy}$ | Δα |
|---|---|---|---|---|---|---|---|---|
| 1ash.pdb | 1ash_1hda-b_r.pdb | 2.993 | -51.74 | -32.54 | -19.2 | -57.08 | -34.51 | -22.57 |
| 1ash.pdb | 1ash_2pgh-a_r.pdb | 3.163 | -51.74 | -33.78 | -17.96 | -57.08 | -34.88 | -22.2 |
| 1ash.pdb | 1ash_4sdh-a_r.pdb | 3.37 | -51.74 | -34 | -17.74 | -57.08 | -35.26 | -21.82 |
| 1ash.pdb | 1ash_1cpc-a_r.pdb | 5.366 | -51.74 | -32.97 | -18.77 | -57.08 | -35.42 | -21.66 |
| 1ash.pdb | 1ash_1hbh-b_r.pdb | 2.979 | -51.74 | -36.27 | -15.47 | -57.08 | -37.06 | -20.02 |
| 1bab-B.pdb | 1bab-b_1cpc-a_r.pdb | 6.527 | -42.35 | -22.73 | -19.62 | -42.22 | -18.42 | -23.8 |
| 1bab-B.pdb | 1bab-b_4sdh-a_r.pdb | 4.957 | -42.35 | -25.95 | -16.4 | -42.22 | -23.25 | -18.97 |
| 1bab-B.pdb | 1bab-b_1flp_r.pdb | 3.673 | -42.35 | -26.46 | -15.89 | -42.22 | -24.29 | -17.93 |
| 1bab-B.pdb | 1bab-b_1ith-a_r.pdb | 4.147 | -42.35 | -27.71 | -14.64 | -42.22 | -24.84 | -17.38 |
| 1bab-B.pdb | 1bab-b_1ecd_r.pdb | 4.296 | -42.35 | -27.45 | -14.9 | -42.22 | -26.38 | -15.84 |
| 1col-A.pdb | 1col-a_4sdh-a_r.pdb | 17.006 | -40.73 | -16.02 | -24.71 | -32.73 | -11.94 | -20.79 |
| 1col-A.pdb | 1col-a_1ith-a_r.pdb | 18.823 | -40.73 | -18.05 | -22.68 | -32.73 | -13.53 | -19.2 |
| 1col-A.pdb | 1col-a_1hbg_r.pdb | 22.865 | -40.73 | -17.8 | -22.93 | -32.73 | -13.62 | -19.11 |
| 1col-A.pdb | 1col-a_1hda-a_r.pdb | 19.906 | -40.73 | -17.26 | -23.47 | -32.73 | -13.9 | -18.83 |
| 1col-A.pdb | 1col-a_1mba_r.pdb | 21.864 | -40.73 | -17.46 | -23.27 | -32.73 | -14.54 | -18.19 |
| 1cpc-A.pdb | 1cpc-a_1hlb_r.pdb | 8.624 | -37.9 | -18.96 | -18.94 | -39.63 | -12.87 | -26.76 |
| 1cpc-A.pdb | 1cpc-a_2pgh-b_r.pdb | 7.568 | -37.9 | -19.57 | -18.33 | -39.63 | -14.25 | -25.38 |
| 1cpc-A.pdb | 1cpc-a_1ash_r.pdb | 9.675 | -37.9 | -18.91 | -18.99 | -39.63 | -14.84 | -24.79 |
| 1cpc-A.pdb | 1cpc-a_2lhb_r.pdb | 13.368 | -37.9 | -19.64 | -18.26 | -39.63 | -15.43 | -24.2 |
| 1cpc-A.pdb | 1cpc-a_1mbs_r.pdb | 10.385 | -37.9 | -21.04 | -16.86 | -39.63 | -16.74 | -22.89 |
| 1ecd.pdb | 1ecd_1cpc-a_r.pdb | 6.188 | -45.99 | -23.98 | -22.01 | -52.49 | -21.87 | -30.62 |
| 1ecd.pdb | 1ecd_1col-a_r.pdb | 4.548 | -45.99 | -22.55 | -23.44 | -52.49 | -23.73 | -28.76 |
| 1ecd.pdb | 1ecd_1ash_r.pdb | 2.638 | -45.99 | -32.1 | -13.89 | -52.49 | -32.59 | -19.9 |
| 1ecd.pdb | 1ecd_1ith-a_r.pdb | 3.263 | -45.99 | -32.81 | -13.18 | -52.49 | -33.3 | -19.19 |
| 1ecd.pdb | 1ecd_1hlm_r.pdb | 2.166 | -45.99 | -33.31 | -12.68 | -52.49 | -35.81 | -16.68 |
| 1emy.pdb | 1emy_1cpc-a_r.pdb | 8.229 | -45.6 | -23.33 | -22.27 | -49.38 | -21 | -28.38 |
| 1emy.pdb | 1emy_1ecd_r.pdb | 6.79 | -45.6 | -24.57 | -21.03 | -49.38 | -24.1 | -25.28 |
| 1emy.pdb | 1emy_2dhb-a_r.pdb | 4.298 | -45.6 | -26.17 | -19.43 | -49.38 | -25.52 | -23.86 |
| 1emy.pdb | 1emy_1bab-a_r.pdb | 6.44 | -45.6 | -26.62 | -18.98 | -49.38 | -25.53 | -23.85 |
| 1emy.pdb | 1emy_1flp_r.pdb | 5.474 | -45.6 | -27.01 | -18.59 | -49.38 | -26.58 | -22.8 |
| 1flp.pdb | 1flp_1cpc-a_r.pdb | 5.63 | -42.56 | -26.4 | -16.16 | -47.42 | -24.39 | -23.03 |
| 1flp.pdb | 1flp_4sdh-a_r.pdb | 3.643 | -42.56 | -28.58 | -13.98 | -47.42 | -27.88 | -19.54 |
| 1flp.pdb | 1flp_1mbs_r.pdb | 2.168 | -42.56 | -31.58 | -10.98 | -47.42 | -30.58 | -16.84 |
| 1flp.pdb | 1flp_1hbg_r.pdb | 2.592 | -42.56 | -30.59 | -11.97 | -47.42 | -30.88 | -16.54 |
| 1flp.pdb | 1flp_1ith-a_r.pdb | 2.206 | -42.56 | -31.55 | -11.01 | -47.42 | -30.92 | -16.5 |
| 1hbg.pdb | 1hbg_2lhb_r.pdb | 4.506 | -37.02 | -26.94 | -10.08 | -39.1 | -23.92 | -15.18 |
| 1hbg.pdb | 1hbg_1cpc-a_r.pdb | 6.896 | -37.02 | -28.35 | -8.67 | -39.1 | -24.24 | -14.86 |
| 1hbg.pdb | 1hbg_4sdh-a_r.pdb | 5.266 | -37.02 | -26.73 | -10.29 | -39.1 | -24.36 | -14.74 |
| 1hbg.pdb | 1hbg_1mba_r.pdb | 2.8 | -37.02 | -27.04 | -9.98 | -39.1 | -25.2 | -13.9 |
| 1hbg.pdb | 1hbg_1col-a_r.pdb | 4.928 | -37.02 | -26.41 | -10.61 | -39.1 | -25.31 | -13.79 |

| | | | | | | | | |
|---|---|---|---|---|---|---|---|---|
| 1hbh-A.pdb | 1hbh-a_1cpc-a_r.pdb | 6.347 | -42.64 | -22.77 | -19.87 | -41.66 | -18.32 | -23.34 |
| 1hbh-A.pdb | 1hbh-a_1ecd_r.pdb | 4.864 | -42.64 | -25.53 | -17.11 | -41.66 | -22.77 | -18.89 |
| 1hbh-A.pdb | 1hbh-a_1ith-a_r.pdb | 4.199 | -42.64 | -27.39 | -15.25 | -41.66 | -23.55 | -18.11 |
| 1hbh-A.pdb | 1hbh-a_1flp_r.pdb | 3.632 | -42.64 | -28.84 | -13.8 | -41.66 | -26.11 | -15.55 |
| 1hbh-A.pdb | 1hbh-a_1ash_r.pdb | 3.809 | -42.64 | -27.65 | -14.99 | -41.66 | -26.19 | -15.47 |
| 1hlb.pdb | 1hlb_1cpc-a_r.pdb | 6.766 | -40.36 | -29.16 | -11.2 | -41.83 | -27.46 | -14.37 |
| 1hlb.pdb | 1hlb_1col-a_r.pdb | 7.001 | -40.36 | -28.74 | -11.62 | -41.83 | -27.92 | -13.91 |
| 1hlb.pdb | 1hlb_4sdh-a_r.pdb | 4.954 | -40.36 | -30.78 | -9.58 | -41.83 | -28.34 | -13.49 |
| 1hlb.pdb | 1hlb_1ith-a_r.pdb | 6.537 | -40.36 | -31.38 | -8.98 | -41.83 | -30.6 | -11.23 |
| 1hlb.pdb | 1hlb_1gdm_r.pdb | 4.95 | -40.36 | -31.81 | -8.55 | -41.83 | -31.35 | -10.48 |
| 1hsy.pdb | 1hsy_1ash_r.pdb | 5.805 | -46.23 | -29.33 | -16.9 | -49.35 | -26.26 | -23.09 |
| 1hsy.pdb | 1hsy_2dhb-b_r.pdb | 4.519 | -46.23 | -36.03 | -10.2 | -49.35 | -34.03 | -15.32 |
| 1hsy.pdb | 1hsy_1hbh-b_r.pdb | 4.293 | -46.23 | -32.07 | -14.16 | -49.35 | -31.32 | -18.03 |
| 1hsy.pdb | 1hsy_1mba_r.pdb | 6.997 | -46.23 | -26.75 | -19.48 | -49.35 | -24.94 | -24.41 |
| 1hsy.pdb | 1hsy_1flp_r.pdb | 6.356 | -46.23 | -27.1 | -19.13 | -49.35 | -25.85 | -23.5 |
| 1ith-A.pdb | 1ith-a_1col-a_r.pdb | 6.071 | -39.48 | -21.09 | -18.39 | -38.64 | -16.58 | -22.06 |
| 1ith-A.pdb | 1ith-a_1cpc-a_r.pdb | 5.107 | -39.48 | -22.21 | -17.27 | -38.64 | -21.96 | -16.68 |
| 1ith-A.pdb | 1ith-a_1gdm_r.pdb | 2.806 | -39.48 | -28.71 | -10.77 | -38.64 | -26.47 | -12.17 |
| 1ith-A.pdb | 1ith-a_2pgh-b_r.pdb | 1.999 | -39.48 | -29.33 | -10.15 | -38.64 | -27.63 | -11.01 |
| 1ith-A.pdb | 1ith-a_1hbh-a_r.pdb | 2.334 | -39.48 | -28.36 | -11.12 | -38.64 | -27.73 | -10.91 |
| 1mba.pdb | 1mba_1cpc-a_r.pdb | 5.956 | -45.7 | -23.55 | -22.15 | -49.34 | -21.89 | -27.45 |
| 1mba.pdb | 1mba_1col-a_r.pdb | 7.314 | -45.7 | -23.42 | -22.28 | -49.34 | -22.26 | -27.08 |
| 1mba.pdb | 1mba_1ith-a_r.pdb | 3.584 | -45.7 | -31.96 | -13.74 | -49.34 | -31.6 | -17.74 |
| 1mba.pdb | 1mba_4sdh-a_r.pdb | 5.02 | -45.7 | -32.67 | -13.03 | -49.34 | -32.96 | -16.38 |
| 1mba.pdb | 1mba_1gdm_r.pdb | 2.859 | -45.7 | -33.21 | -12.49 | -49.34 | -34.66 | -14.68 |
| 1mbs.pdb | 1mbs_1cpc-a_r.pdb | 7.509 | -28.82 | -21.38 | -7.44 | -32.22 | -20.02 | -12.2 |
| 1mbs.pdb | 1mbs_1ecd_r.pdb | 7.339 | -28.82 | -22.49 | -6.33 | -32.22 | -22.47 | -9.75 |
| 1mbs.pdb | 1mbs_1ash_r.pdb | 6.656 | -28.82 | -26.4 | -2.42 | -32.22 | -25.2 | -7.02 |
| 1mbs.pdb | 1mbs_1hbg_r.pdb | 5.796 | -28.82 | -29.56 | 0.74 | -32.22 | -27.14 | -5.08 |
| 1mbs.pdb | 1mbs_1col-a_r.pdb | 7.903 | -28.82 | -29.14 | 0.32 | -32.22 | -30.11 | -2.11 |

| **Natives** | **Decoys**[1] | **RMSD** | **$Gen_{Native}$** | **$Gen_{Decoy}$** | **$\Delta Gen$** | **$\beta_{Native}$** | **$\beta_{Decoy}$** | **$\Delta\beta$** |
|---|---|---|---|---|---|---|---|---|
| 1acy.pdb | 1acy_FV:_on_1yuh_FV:_r.pdb | 4.338 | -30.04 | -19.81 | -10.23 | -20.43 | -6.98 | -13.45 |
| 1acy.pdb | 1acy_FV:_on_1mfa_FV:_r.pdb | 3.669 | -30.04 | -20.82 | -9.22 | -20.43 | -8.71 | -11.72 |
| 1acy.pdb | 1acy_FV:_on_1ind_FV:_r.pdb | 2.403 | -30.04 | -26.13 | -3.91 | -20.43 | -14.97 | -5.46 |
| 1acy.pdb | 1acy_FV:_on_1hkl_FV:_r.pdb | 1.741 | -30.04 | -25.03 | -5.01 | -20.43 | -15.39 | -5.04 |
| 1acy.pdb | 1acy_FV:_on_1mam_FV:_r.pdb | 3.136 | -30.04 | -25.68 | -4.36 | -20.43 | -15.53 | -4.9 |
| 1baf.pdb | 1baf_FV:_on_1yuh_FV:_r.pdb | 3.919 | -27.82 | -20.39 | -7.43 | -17.69 | -6.39 | -11.3 |
| 1baf.pdb | 1baf_FV:_on_1mfa_FV:_r.pdb | 3.13 | -27.82 | -20.25 | -7.57 | -17.69 | -6.9 | -10.79 |
| 1baf.pdb | 1baf_FV:_on_7fab_FV:_r.pdb | 3.168 | -27.82 | -24.69 | -3.13 | -17.69 | -12.53 | -5.16 |
| 1baf.pdb | 1baf_FV:_on_1gaf_FV:_r.pdb | 1.772 | -27.82 | -25.43 | -2.39 | -17.69 | -13.49 | -4.2 |
| 1baf.pdb | 1baf_FV:_on_1ibg_FV:_r.pdb | 3.719 | -27.82 | -25.24 | -2.58 | -17.69 | -14.6 | -3.09 |
| 1bbd.pdb | 1bbd_FV:_on_1mfa_FV:_r.pdb | 3.918 | -31.17 | -19.52 | -11.65 | -23.36 | -9.34 | -14.02 |
| 1bbd.pdb | 1bbd_FV:_on_1yuh_FV:_r.pdb | 4.537 | -31.17 | -18.9 | -12.27 | -23.36 | -9.46 | -13.9 |

| | | | | | | | | |
|---|---|---|---|---|---|---|---|---|
| 1bbd.pdb | 1bbd_FV:_on_7fab_FV:_r.pdb | 3.633 | -31.17 | -27.47 | -3.7 | -23.36 | -17.3 | -6.06 |
| 1bbd.pdb | 1bbd_FV:_on_1gig_FV:_r.pdb | 2.571 | -31.17 | -27.46 | -3.71 | -23.36 | -19.05 | -4.31 |
| 1bbd.pdb | 1bbd_FV:_on_1ind_FV:_r.pdb | 2.798 | -31.17 | -27.59 | -3.58 | -23.36 | -19.16 | -4.2 |
| 1bbj.pdb | 1bbj_FV:_on_1mfa_FV:_r.pdb | 3.222 | -29.89 | -21.77 | -8.12 | -25.25 | -14.01 | -11.24 |
| 1bbj.pdb | 1bbj_FV:_on_1yuh_FV:_r.pdb | 4.047 | -29.89 | -20.79 | -9.1 | -25.25 | -14.05 | -11.2 |
| 1bbj.pdb | 1bbj_FV:_on_7fab_FV:_r.pdb | 3.232 | -29.89 | -28.61 | -1.28 | -25.25 | -22.63 | -2.62 |
| 1bbj.pdb | 1bbj_FV:_on_1gaf_FV:_r.pdb | 1.116 | -29.89 | -29.81 | -0.08 | -25.25 | -23.84 | -1.41 |
| 1bbj.pdb | 1bbj_FV:_on_1ngq_FV:_r.pdb | 1.737 | -29.89 | -31.1 | 1.21 | -25.25 | -24.06 | -1.19 |
| 1dbb.pdb | 1dbb_FV:_on_1yuh_FV:_r.pdb | 4.138 | -27.64 | -16.47 | -11.17 | -15.62 | -1.34 | -14.28 |
| 1dbb.pdb | 1dbb_FV:_on_1mfa_FV:_r.pdb | 3.5 | -27.64 | -18.4 | -9.24 | -15.62 | -3.08 | -12.54 |
| 1dbb.pdb | 1dbb_FV:_on_1gig_FV:_r.pdb | 2.293 | -27.64 | -25.61 | -2.03 | -15.62 | -10.86 | -4.76 |
| 1dbb.pdb | 1dbb_FV:_on_1baf_FV:_r.pdb | 1.843 | -27.64 | -25.42 | -2.22 | -15.62 | -11.99 | -3.63 |
| 1dbb.pdb | 1dbb_FV:_on_2fb4_FV:_r.pdb | 2.171 | -27.64 | -25.61 | -2.03 | -15.62 | -12.61 | -3.01 |
| 1dfb.pdb | 1dfb_FV:_on_1yuh_FV:_r.pdb | 4.354 | -28.8 | -17.34 | -11.46 | -20.6 | -5.97 | -14.63 |
| 1dfb.pdb | 1dfb_FV:_on_1mfa_FV:_r.pdb | 3.688 | -28.8 | -18.06 | -10.74 | -20.6 | -6.29 | -14.31 |
| 1dfb.pdb | 1dfb_FV:_on_1mam_FV:_r.pdb | 2.4 | -28.8 | -24.86 | -3.94 | -20.6 | -15.2 | -5.4 |
| 1dfb.pdb | 1dfb_FV:_on_3hfm_FV:_r.pdb | 1.96 | -28.8 | -26.35 | -2.45 | -20.6 | -16.31 | -4.29 |
| 1dfb.pdb | 1dfb_FV:_on_1gaf_FV:_r.pdb | 4.826 | -28.8 | -24.1 | -4.7 | -20.6 | -16.38 | -4.22 |
| 1dvf.pdb | 1dvf_FV:_on_1yuh_FV:_r.pdb | 4.118 | -31.46 | -21.32 | -10.14 | -24.75 | -11.54 | -13.21 |
| 1dvf.pdb | 1dvf_FV:_on_1mfa_FV:_r.pdb | 3.261 | -31.46 | -21.52 | -9.94 | -24.75 | -11.88 | -12.87 |
| 1dvf.pdb | 1dvf_FV:_on_1ind_FV:_r.pdb | 1.891 | -31.46 | -27.58 | -3.88 | -24.75 | -19.26 | -5.49 |
| 1dvf.pdb | 1dvf_FV:_on_2gfb_FV:_r.pdb | 3.063 | -31.46 | -28.33 | -3.13 | -24.75 | -19.91 | -4.84 |
| 1dvf.pdb | 1dvf_FV:_on_1baf_FV:_r.pdb | 1.465 | -31.46 | -27.97 | -3.49 | -24.75 | -20.11 | -4.64 |
| 1eap.pdb | 1eap_FV:_on_1mfa_FV:_r.pdb | 3.774 | -30.17 | -20.23 | -9.94 | -24.26 | -12.21 | -12.05 |
| 1eap.pdb | 1eap_FV:_on_1yuh_FV:_r.pdb | 4.537 | -30.17 | -20.24 | -9.93 | -24.26 | -13.15 | -11.11 |
| 1eap.pdb | 1eap_FV:_on_1ucb_FV:_r.pdb | 2.474 | -30.17 | -27.66 | -2.51 | -24.26 | -19.44 | -4.82 |
| 1eap.pdb | 1eap_FV:_on_1ggi_FV:_r.pdb | 2.291 | -30.17 | -26.32 | -3.85 | -24.26 | -19.9 | -4.36 |
| 1eap.pdb | 1eap_FV:_on_1hkl_FV:_r.pdb | 2.425 | -30.17 | -26.93 | -3.24 | -24.26 | -19.91 | -4.35 |
| 1fai.pdb | 1fai_FV:_on_1mfa_FV:_r.pdb | 3.714 | -28.32 | -19.84 | -8.48 | -19.8 | -10.36 | -9.44 |
| 1fai.pdb | 1fai_FV:_on_1yuh_FV:_r.pdb | 4.487 | -28.32 | -19.8 | -8.52 | -19.8 | -10.99 | -8.81 |
| 1fai.pdb | 1fai_FV:_on_2fb4_FV:_r.pdb | 1.833 | -28.32 | -25.44 | -2.88 | -19.8 | -16.69 | -3.11 |
| 1fai.pdb | 1fai_FV:_on_1jel_FV:_r.pdb | 2.253 | -28.32 | -25.36 | -2.96 | -19.8 | -17.22 | -2.58 |
| 1fai.pdb | 1fai_FV:_on_1gaf_FV:_r.pdb | 2.069 | -28.32 | -26.57 | -1.75 | -19.8 | -17.64 | -2.16 |
| 1fbi.pdb | 1fbi_FV:_on_1mfa_FV:_r.pdb | 3.481 | -26.58 | -19.78 | -6.8 | -15.64 | -8.17 | -7.47 |
| 1fbi.pdb | 1fbi_FV:_on_1yuh_FV:_r.pdb | 4.115 | -26.58 | -20.99 | -5.59 | -15.64 | -11.23 | -4.41 |
| 1fbi.pdb | 1fbi_FV:_on_1mam_FV:_r.pdb | 3.481 | -26.58 | -24.51 | -2.07 | -15.64 | -15.05 | -0.59 |
| 1fbi.pdb | 1fbi_FV:_on_1igf_FV:_r.pdb | 1.997 | -26.58 | -25.83 | -0.75 | -15.64 | -15.29 | -0.35 |
| 1fbi.pdb | 1fbi_FV:_on_1hkl_FV:_r.pdb | 1.96 | -26.58 | -26.01 | -0.57 | -15.64 | -15.32 | -0.32 |
| 1fgv.pdb | 1fgv_FV:_on_1mfa_FV:_r.pdb | 3.455 | -31.52 | -19.5 | -12.02 | -23.04 | -9.27 | -13.77 |
| 1fgv.pdb | 1fgv_FV:_on_1yuh_FV:_r.pdb | 4.277 | -31.52 | -20.8 | -10.72 | -23.04 | -10.72 | -12.32 |
| 1fgv.pdb | 1fgv_FV:_on_3hfm_FV:_r.pdb | 1.785 | -31.52 | -26.45 | -5.07 | -23.04 | -17.09 | -5.95 |
| 1fgv.pdb | 1fgv_FV:_on_7fab_FV:_r.pdb | 3.075 | -31.52 | -26.54 | -4.98 | -23.04 | -17.81 | -5.23 |
| 1fgv.pdb | 1fgv_FV:_on_1bbj_FV:_r.pdb | 1.737 | -31.52 | -26.92 | -4.6 | -23.04 | -18.81 | -4.23 |
| 1fig.pdb | 1fig_FV:_on_1mfa_FV:_r.pdb | 3.474 | -23.96 | -18.92 | -5.04 | -18.3 | -10.53 | -7.77 |
| 1fig.pdb | 1fig_FV:_on_1yuh_FV:_r.pdb | 4.364 | -23.96 | -19.99 | -3.97 | -18.3 | -11.8 | -6.5 |

| | | | | | | | | |
|---|---|---|---|---|---|---|---|---|
| 1fig.pdb | 1fig_FV:_on_1gaf_FV:_r.pdb | 2.078 | -23.96 | -24.61 | 0.65 | -18.3 | -17.31 | -0.99 |
| 1fig.pdb | 1fig_FV:_on_1hkl_FV:_r.pdb | 1.809 | -23.96 | -27.13 | 3.17 | -18.3 | -17.92 | -0.38 |
| 1fig.pdb | 1fig_FV:_on_1ibg_FV:_r.pdb | 4.165 | -23.96 | -27.13 | 3.17 | -18.3 | -18.82 | 0.52 |
| 1flr.pdb | 1flr_FV:_on_1yuh_FV:_r.pdb | 4.176 | -29.94 | -19.76 | -10.18 | -19.74 | -6.29 | -13.45 |
| 1flr.pdb | 1flr_FV:_on_1mfa_FV:_r.pdb | 3.72 | -29.94 | -20.43 | -9.51 | -19.74 | -7.5 | -12.24 |
| 1flr.pdb | 1flr_FV:_on_7fab_FV:_r.pdb | 3.355 | -29.94 | -27.63 | -2.31 | -19.74 | -15.71 | -4.03 |
| 1flr.pdb | 1flr_FV:_on_1vge_FV:_r.pdb | 3.975 | -29.94 | -27.46 | -2.48 | -19.74 | -15.76 | -3.98 |
| 1flr.pdb | 1flr_FV:_on_1ggi_FV:_r.pdb | 1.926 | -29.94 | -28 | -1.94 | -19.74 | -16.27 | -3.47 |
| 1for.pdb | 1for_FV:_on_1mfa_FV:_r.pdb | 4.143 | -26.95 | -20.51 | -6.44 | -18.46 | -10.23 | -8.23 |
| 1for.pdb | 1for_FV:_on_1yuh_FV:_r.pdb | 4.162 | -26.95 | -20.51 | -6.44 | -18.46 | -10.52 | -7.94 |
| 1for.pdb | 1for_FV:_on_1hkl_FV:_r.pdb | 1.454 | -26.95 | -26.23 | -0.72 | -18.46 | -16.61 | -1.85 |
| 1for.pdb | 1for_FV:_on_1mam_FV:_r.pdb | 3.166 | -26.95 | -25.49 | -1.46 | -18.46 | -16.67 | -1.79 |
| 1for.pdb | 1for_FV:_on_1flr_FV:_r.pdb | 1.706 | -26.95 | -27.72 | 0.77 | -18.46 | -17.26 | -1.2 |
| 1fpt.pdb | 1fpt_FV:_on_1yuh_FV:_r.pdb | 4.063 | -29.42 | -19.01 | -10.41 | -19.01 | -7.73 | -11.28 |
| 1fpt.pdb | 1fpt_FV:_on_1mfa_FV:_r.pdb | 3.404 | -29.42 | -20.2 | -9.22 | -19.01 | -8.37 | -10.64 |
| 1fpt.pdb | 1fpt_FV:_on_1ind_FV:_r.pdb | 2.315 | -29.42 | -25.18 | -4.24 | -19.01 | -14.23 | -4.78 |
| 1fpt.pdb | 1fpt_FV:_on_1ggi_FV:_r.pdb | 2.092 | -29.42 | -23.89 | -5.53 | -19.01 | -14.41 | -4.6 |
| 1fpt.pdb | 1fpt_FV:_on_2gfb_FV:_r.pdb | 2.225 | -29.42 | -26.41 | -3.01 | -19.01 | -14.53 | -4.48 |
| 1frg.pdb | 1frg_FV:_on_1mfa_FV:_r.pdb | 3.653 | -31.23 | -20.26 | -10.97 | -22.8 | -8.68 | -14.12 |
| 1frg.pdb | 1frg_FV:_on_1yuh_FV:_r.pdb | 4.09 | -31.23 | -19.82 | -11.41 | -22.8 | -8.88 | -13.92 |
| 1frg.pdb | 1frg_FV:_on_1hkl_FV:_r.pdb | 2.403 | -31.23 | -25.38 | -5.85 | -22.8 | -15.72 | -7.08 |
| 1frg.pdb | 1frg_FV:_on_1mlb_FV:_r.pdb | 2.159 | -31.23 | -24.6 | -6.63 | -22.8 | -16.1 | -6.7 |
| 1frg.pdb | 1frg_FV:_on_1ind_FV:_r.pdb | 2.456 | -31.23 | -26.4 | -4.83 | -22.8 | -16.11 | -6.69 |
| 1fvc.pdb | 1fvc_FV:_on_1mfa_FV:_r.pdb | 3.515 | -29.88 | -19.3 | -10.58 | -22.19 | -7.76 | -14.43 |
| 1fvc.pdb | 1fvc_FV:_on_1yuh_FV:_r.pdb | 4.248 | -29.88 | -21.08 | -8.8 | -22.19 | -9.31 | -12.88 |
| 1fvc.pdb | 1fvc_FV:_on_7fab_FV:_r.pdb | 4.97 | -29.88 | -27.14 | -2.74 | -22.19 | -16.75 | -5.44 |
| 1fvc.pdb | 1fvc_FV:_on_1ind_FV:_r.pdb | 4.221 | -29.88 | -28.1 | -1.78 | -22.19 | -17.74 | -4.45 |
| 1fvc.pdb | 1fvc_FV:_on_1baf_FV:_r.pdb | 3.949 | -29.88 | -28.22 | -1.66 | -22.19 | -18.5 | -3.69 |
| 1fvd.pdb | 1fvd_FV:_on_1mfa_FV:_r.pdb | 3.263 | -30.63 | -20.01 | -10.62 | -23.19 | -8.38 | -14.81 |
| 1fvd.pdb | 1fvd_FV:_on_1yuh_FV:_r.pdb | 4.03 | -30.63 | -20 | -10.63 | -23.19 | -9.95 | -13.24 |
| 1fvd.pdb | 1fvd_FV:_on_7fab_FV:_r.pdb | 2.955 | -30.63 | -28.07 | -2.56 | -23.19 | -19.33 | -3.86 |
| 1fvd.pdb | 1fvd_FV:_on_1ind_FV:_r.pdb | 1.673 | -30.63 | -28.2 | -2.43 | -23.19 | -19.38 | -3.81 |
| 1fvd.pdb | 1fvd_FV:_on_2gfb_FV:_r.pdb | 2.297 | -30.63 | -27.38 | -3.25 | -23.19 | -19.62 | -3.57 |

| **Natives** | **Decoys**[1] | **RMSD** | **$Gen_{Native}$** | **$Gen_{Decoy}$** | **ΔGen** | **$\alpha+\beta_{Native}$** | **$\alpha+\beta_{Decoy}$** | **Δ(α+β)** |
|---|---|---|---|---|---|---|---|---|
| 1sn3.pdb | 1sn3.e9387_r.pdb | 3.116 | -28.54 | -9.74 | -18.8 | -29.71 | -10.24 | -19.47 |
| 1sn3.pdb | 1sn3.f5168_r.pdb | 5.139 | -28.54 | -11.07 | -17.47 | -29.71 | -12.45 | -17.26 |
| 1sn3.pdb | 1sn3.f1088_r.pdb | 4.122 | -28.54 | -14.53 | -14.01 | -29.71 | -14.29 | -15.42 |
| 1sn3.pdb | 1sn3.b4755_r.pdb | 2.153 | -28.54 | -18.39 | -10.15 | -29.71 | -18.53 | -11.18 |
| 1sn3.pdb | 1sn3.a7566_r.pdb | 1.31 | -28.54 | -20.54 | -8 | -29.71 | -21.69 | -8.02 |
| 4pti.pdb | 4pti.e16495_r.pdb | 4.106 | -26.75 | -10.41 | -16.34 | -28.44 | -11.86 | -16.58 |
| 4pti.pdb | 4pti.e3771_r.pdb | 5.178 | -26.75 | -18.85 | -7.9 | -28.44 | -18.69 | -9.75 |
| 4pti.pdb | 4pti.c9594_r.pdb | 2.125 | -26.75 | -17.79 | -8.96 | -28.44 | -18.93 | -9.51 |
| 4pti.pdb | 4pti.c20227_r.pdb | 1.414 | -26.75 | -19.11 | -7.64 | -28.44 | -20.29 | -8.15 |

| | | | | | | | | |
|---|---|---|---|---|---|---|---|---|
| 4pti.pdb | 4pti.d6727_r.pdb | 3.126 | -26.75 | -21.49 | -5.26 | -28.44 | -22.07 | -6.37 |
| 1ctf.pdb | 1ctf.65675.pdb | 4.129 | -45.23 | -33.19 | -12.04 | -42.83 | -30.74 | -12.09 |
| 1ctf.pdb | 1ctf.35240.pdb | 5.188 | -45.23 | -33.81 | -11.42 | -42.83 | -31.71 | -11.12 |
| 1ctf.pdb | 1ctf.65079.pdb | 4.246 | -45.23 | -37.13 | -8.1 | -42.83 | -35.01 | -7.82 |
| 1ctf.pdb | 1ctf.728.pdb | 4.377 | -45.23 | -37.85 | -7.38 | -42.83 | -35.09 | -7.74 |
| 1ctf.pdb | 1ctf.41701.pdb | 3.905 | -45.23 | -43.09 | -2.14 | -42.83 | -40.89 | -1.94 |
| 4icb.pdb | akcalb55-min.pdb | 5.269 | -50.02 | -30.11 | -19.91 | -47.75 | -28.72 | -19.03 |
| 4icb.pdb | apcalb74-min.pdb | 4.754 | -50.02 | -35.86 | -14.16 | -47.75 | -34.26 | -13.49 |
| 4icb.pdb | akcalb98-min.pdb | 5.271 | -50.02 | -39.91 | -10.11 | -47.75 | -37.6 | -10.15 |
| 4icb.pdb | akcalb63-min.pdb | 5.39 | -50.02 | -41.83 | -8.19 | -47.75 | -39.8 | -7.95 |
| 4icb.pdb | akcalb50-min.pdb | 4.912 | -50.02 | -42.25 | -7.77 | -47.75 | -40.3 | -7.45 |

Note: $Gen_{native}$ and $Gen_{Decoy}$ are the RAPDF scores for native and decoy proteins using the general statistical library; $\alpha_{native}$ and $\alpha_{Decoy}$ are the RAPDF scores for native and decoy proteins using the α statistical library; $\beta_{native}$ and $\beta_{Decoy}$ are the RAPDF scores for native and decoy proteins using the β statistical library; $\alpha+\beta_{native}$ and $\alpha+\beta_{Decoy}$ are the RAPDF scores for native and decoy proteins using the α+β statistical library. Δ represents the difference in the RAPDF scores between the native and decoy proteins. All decoy proteins are from the Decoy R Us website by Samudrala R. (The names of the sets for α, β and α+β are hg_structural, ig_structural, 4state_reduced&lmds, respectively [1])

**Reference:**


1. Samudrala R, Levitt M. Decoys 'R' Us: A database of incorrect protein conformations to improve protein structure prediction. Protein Science. 2000; 9: 1399-1401.
