# Supplementary material for: StaRProtein, A Web Server for Prediction of the Stability of Repeat Proteins
Source: PLoS One. 2015 Mar 25;10(3):e0119417. doi: 10.1371/journal.pone.0119417 (PMC4373711; doi:10.1371/journal.pone.0119417)
Supplement: S2 Table — (PDF) [file pone.0119417.s005.pdf]

S2 Table

Comparison of kinetic energies and RAPDF scores of globular proteins.

	PDB code	Urea (ΔG_D^{H2O}) (kcal/mol)	m	Gdn-HCl (ΔG_D^{H2O}) (kcal/mol)	m	RAPDF (General)
**Rnase A [1]**	7RSA	7.4	1.14	7.5	2.5	-33.71
**Lysozyme [1]**	132L	8.8	1.29	8.9	1.88	-35.3
**α-Lactalbumin [1]**	1A4V	4.4	0.88	4.2	1.27	-37.04
**Myoglobin [1]**	IMBO	8.1	2.08	7.6	4.22	-47.57
**L7Ae [2]**	2FC3			12 ± 3	2 ± 0.5	-43.54

References

1. Ahmad F, Bigelow CC. Estimation of the free energy of stabilization of ribonuclease A, lysozyme, alpha-lactalbumin, and myoglobin. J Biol Chem. 1982; 257:12935-8.
2. Bhuiya MW, Suryadi J, Zhou Z, Brown BA 2nd. Structure of the Aeropyrum pernix L7Ae multifunctional protein and insight into its extreme thermostability. Acta Crystallogr Sect F Struct Biol Cryst Commun. 2013; 69:979-88.
